# A mobile loop near the active site acts as a switch between the dual activities of a viral protease/deubiquitinase

**DOI:** 10.1371/journal.ppat.1006714

**Published:** 2017-11-08

**Authors:** Isabelle Jupin, Maya Ayach, Lucile Jomat, Sonia Fieulaine, Stéphane Bressanelli

**Affiliations:** 1 Institut Jacques Monod, CNRS—Univ Paris-Diderot, Paris, France; 2 Institute for Integrative Biology of the Cell, CEA—CNRS—Univ Paris-Saclay, Gif sur Yvette, France; Purdue University, UNITED STATES

## Abstract

The positive-strand RNA virus *Turnip yellow mosaic virus* (TYMV) encodes an ovarian tumor (OTU)-like protease/deubiquitinase (PRO/DUB) protein domain involved both in proteolytic processing of the viral polyprotein through its PRO activity, and in removal of ubiquitin chains from ubiquitylated substrates through its DUB activity. Here, the crystal structures of TYMV PRO/DUB mutants and molecular dynamics simulations reveal that an idiosyncratic mobile loop participates in reversibly constricting its unusual catalytic site by adopting "open", "intermediate" or "closed" conformations. The two *cis*-prolines of the loop form a rigid flap that in the most closed conformation zips up against the other side of the catalytic cleft. The intermediate and closed conformations also correlate with a reordering of the TYMV PRO/DUB catalytic dyad, that then assumes a classical, yet still unusually mobile, OTU DUB alignment. Further structure-based mutants designed to interfere with the loop's mobility were assessed for enzymatic activity *in vitro* and *in vivo*, and were shown to display reduced DUB activity while retaining PRO activity. This indicates that control of the switching between the dual PRO/DUB activities resides prominently within this loop next to the active site. Introduction of mutations into the viral genome revealed that the DUB activity contributes to the extent of viral RNA accumulation both in single cells and in whole plants. In addition, the conformation of the mobile flap was also found to influence symptoms severity *in planta*. Such mutants now provide powerful tools with which to study the specific roles of reversible ubiquitylation in viral infection.

## Author summary

Viruses have much smaller genomes than their hosts. Consequently, they often encode proteins which are multifunctional. For instance, some viral proteases have a dual function, being also deubiquitinases, i.e. enzymes capable of removing ubiquitin tags grafted onto proteins and that often target them for destruction. The protease and deubiquitinase activities share a single active site that is used alternately for one function or the other, but how this switch between activities may be regulated is presently unknown. To answer this question, we studied a simple plant virus that is a useful model system for these complex molecular biology phenomena, and that encodes a simplified protease/deubiquitinase. Here, thanks to a combination of structural and functional analyses, we managed to decouple the two activities, killing the deubiquitinase activity while preserving the protease one. This successful decoupling relies on our discovery that a loop inserted next to the active site is mobile, and can thus act as a switch between the two activities. This result allowed us to demonstrate the importance of the specific deubiquinase activity in viral multiplication. In addition, viral symptoms were also severely affected by mutations affecting the loop mobility. Our data provide powerful tools for further studies, that may also be relevant for more complex or medically relevant viruses.

## Introduction

Ubiquitin (Ub) is a 76-residue protein that is highly conserved throughout the eukaryotic kingdom. Attachment of Ub to cellular proteins (referred to as ubiquitylation) is recognized as a key regulatory pathway, critical to a number of major cellular processes, including protein homeostasis, intracellular signalling, transcription, and immune responses [1].

Ub is covalently linked to the target protein via an isopeptide bond between its C-terminal Gly residue, and an acceptor amino acid of the target protein substrate, in most cases a Lys residue. Further conjugation steps to Ub itself can generate polyubiquitylated chains in a number of structurally different configurations [2, 3] that may play different roles in the cell, illustrating the exquisite versatility of Ub conjugation [4, 5].

Importantly, ubiquitylation is a dynamic process that can be reversed by the action of enzymes known as Ub hydrolases, deubiquitinases or deubiquitylating enzymes (DUBs) [6, 7]. Most of these enzymes are Cys proteinases, which can either trim or remove poly-Ub chains from substrate proteins, thus contributing to the reversal of Ub-dependent processes in cells. The breadth of DUBs in the regulation of cellular processes, and their role in promoting human disease, has become apparent over recent years [8, 9], leading to further insights into the mechanistic details of such enzymes and their regulation, combined with increased interest in their use as potential drug targets [10, 11].

It has become increasingly clear that the involvement of ubi- and deubiquitylation events also extends to the regulation of interactions between hosts and pathogens. The ubiquitin proteasome system (UPS) is utilized not only by host cells in immune and biotic stress responses [12, 13], but can also be manipulated and subverted by pathogens—including viruses—for their own use [14–17]. Viruses use various strategies to exploit Ub and Ub-like modifier pathways, with some recruiting host enzymes, whereas others encode their own ubiquitin ligases, or their own DUBs. Such mechanisms may be beneficial to the virus either by creating a more favorable cellular environment, by fine-tuning viral regulatory processes, or by inhibiting host defense mechanisms.

Although important insights into the biochemical activities and molecular structures of viral-encoded DUB enzymes have been gained in recent years [18–23], a major challenge in this field remains to define and understand enzyme specificity and regulation—both towards the various Ub-chain types or target protein substrates, as well as the physiological roles played by these modulators of the Ub pathway during the infection cycle.

This is particularly challenging for viruses encoding a DUB activity in a multifunctional enzyme, as the respective contributions to DUB and other functions are not easily disentangled. In particular, several viral DUBs encoded by positive-strand (+)RNA viruses appear to have dual activities, as they were originally described as being papain-like cysteine proteases (PRO), whose endopeptidase activity is involved in the processing of viral precursor proteins [24, 25]. Both PRO and DUB activities rely on the single catalytic site of the cysteine proteinase, but the determinants regulating these dual endo- and iso-peptidase activities remain largely unknown.

In this paper, using *Turnip yellow mosaic virus* (TYMV), the type member of the genus *Tymovirus*, we address the question of how a viral PRO/DUB enzyme can switch from one activity to the other.

TYMV is a simple model of a (+)RNA virus that is well characterized at the molecular and cellular levels, and whose replication cycle appears to involve reversible ubiquitylation events. We previously reported that TYMV-encoded proteins—including the viral RNA-dependent RNA polymerase—are targets of the UPS *in vitro* and *in vivo* [26–28]. We also reported that the TYMV papain-like cysteine PRO domain involved in the proteolytic processing of the viral replication precursor protein [29–31], also displays a DUB activity that was able to rescue the viral polymerase from degradation [32].

The structure of the recombinant TYMV PRO/DUB domain we solved previously (residues 728–879 of the TYMV replication precursor protein) [21] highlighted its homology with members of the ovarian tumor domain-containing (OTU) superfamily of DUBs [33]. However, TYMV PRO/DUB appeared to be a very peculiar OTU DUB, both functionally and structurally. Indeed, in contrast to other OTU DUBs, TYMV PRO/DUB does not possess a general deubiquitylating activity, but instead displays a specificity towards particular ubiquitylated substrates, among them TYMV RNA-dependent RNA polymerase [32]. In addition, the TYMV PRO/DUB structure displayed a surprisingly exposed and minimal catalytic site, with a Cys783-His869 catalytic dyad [21] instead of the conserved OTU DUB Cys-His-Asp/Asn catalytic triad [6, 34, 35].

In TYMV polyprotein processing, one endopeptidase site lies at the C-terminus of PRO/DUB itself [31] (S1 Fig), and, in our previously described crystal structure [21], each molecule was found in complex with the neighbouring molecule, with its five C-terminal residues inserted into the catalytic cleft of the next molecule. Thus, a product state of polyprotein processing activity was actually captured in that crystal. This PRO:PRO complex revealed the structural basis of extensive recognition of protein substrates on the so called "P side" [36], i.e. residues upstream of the scissile bond. In contrast, the exposed nature of the catalytic dyad suggested that interactions may be rather limited on the P' side, i.e. downstream of the cleavage site.

The crystal structure also allowed us to identify several structural elements, distant from the active site, that are involved in the interaction of PRO/DUB with itself, and possibly with its other substrate partners such as Ub. Among them, a hydrophobic patch some 17 Å away from the active site was used by the cleaving PRO to recognize the cleaved PRO. *In silico* modeling of PRO/DUB:Ub complexes [21] suggested that Ub chains could also be recognized by this distant hydrophobic patch in addition to the interactions in and around the catalytic site. The involvement of such distant Ub recognition patches is now established as a prominent feature among viral and cellular DUBs [22, 23, 37].

In addition, the PRO:PRO complex also engaged several elements close to the active site, in particular a loop in the cleaved PRO that represents an insertion with respect to the closest OTU DUB relatives of TYMV PRO/DUB. This loop, conserved in *Tymoviridae*, bears an atypical 865-GPP-867 motif, that the structure revealed as having both prolines in *cis* conformation [21]. Because this motif is directly upstream of the catalytic His869, we could not rule out the possibility that such interactions may account, in part, for the unusually disordered state of the Cys783-His869 catalytic dyad.

To further understand the molecular, and possibly mechanistic, processes underlying the dual activities of the TYMV PRO/DUB enzyme, we extended our earlier structural analysis by obtaining the structure of a disengaged TYMV PRO/DUB domain, *i*.*e*. one not involved in a PRO:PRO complex. For this purpose, two mutant versions of TYMV PRO/DUB designed to weaken PRO:PRO interactions were expressed and crystallized [38]. The structures reported herein reveal that the loop harboring the 865-GPP-867 motif is a highly mobile flap on the otherwise rigid PRO/DUB framework a result that is confirmed by molecular dynamics simulations. Closure of this flap brings the catalytic site region closer to the canonical OTU DUB active site as seen in complex with Ub, even though the catalytic dyad remains exposed and unusually flexible. Functional analyses of structure-guided mutants indicate that the GPP flap acts as a switch for DUB activity, making it possible to dissociate the PRO and DUB activities of the enzyme, and to analyze their function in the viral life cycle independently. Viruses carrying these mutations displayed a reduced replication efficiency, confirming the critical importance of the DUB activity of PRO/DUB in control of the TYMV replication cycle, independently of the polyprotein processing. In addition, we observed that mutations affecting the loop mobility also markedly affected the severity of viral symptoms *in planta*.

The present study thus provides new structural insights into the mechanisms by which viral OTU-like enzymes with dual PRO/DUB activities can switch between these two activities, and also generates powerful tools with which to further study the importance of solely the DUB activity in virus/host interactions.

## Results

### The crystal structure of C-terminally truncated TYMV PRO/DUB reveals a mobile loop, closure of which constricts the P side of the active site

In order to get an atomic view of a conformation of PRO/DUB not constrained by the crystal-induced PRO:PRO interaction, we generated a recombinant mutant harbouring deletion of the five C-terminal residues (hereafter ΔC5), corresponding to residues 728–874 of the TYMV 206K polyprotein. Crystals of ΔC5 diffracting to beyond 2 Å resolution were obtained [38], and we have since collected data extending to beyond 1.7 Å resolution. We therefore used these new data to solve the ΔC5 structure by molecular replacement from WT PRO (Table 1).

**Table 1:** Crystallographic data collection and refinement statistics.

	ΔC5	I847A
Wavelength		
Resolution range (Å)	36.43–1.649 (1.708–1.649)	36.34–1.653 (1.712–1.653)
Space group	C 1 2 1	C 1 2 1
Unit cell	133.46 39.695 72.864 90 122.127 90	132.722 39.677 72.699 90 121.974 90
Total reflections	140,953 (12,263)	140,017 (12,381)
Unique reflections	39,006 (3797)	38,202 (3525)
Multiplicity	3.6 (3.2)	3.7 (3.5)
Completeness (%)	0.99 (0.98)	0.98 (0.93)
Mean I/sigma(I)	11.22 (1.33)	11.81 (1.51)
Wilson B-factor	23.95	23.99
R-merge	0.06178 (0.6237)	0.06302 (0.9332)
R-meas	0.07265 (0.7481)	0.07389 (1.098)
CC1/2	0.998 (0.909)	0.998 (0.711)
CC*	1 (0.976)	0.999 (0.912)
Reflections used in refinement	38,839 (3762)	38,164 (3510)
Reflections used for R-free	2973 (284)	2928 (265)
R-work	0.2003 (0.4066)	0.2003 (0.4066)
R-free	0.2345 (0.4328)	0.2345 (0.4328)
CC(work)	0.963 (0.911)	0.963 (0.911)
CC(free)	0.952 (0.885)	0.952 (0.885)
Number of non-hydrogen atoms	2437	2437
Macromolecules	2287	2287
Protein residues	288	293
RMS (bonds)	0.005	0.006
RMS (angles)	0.81	0.84
Ramachandran favored (%)	99	99
Ramachandran allowed (%)	0.69	1.0
Ramachandran outliers (%)	0	0
Rotamer outliers (%)	0.69	1.0
Clashscore	1.54	3.47
Average B-factor	38.65	35.77
Macromolecules (A / B / both)	34.1 / 43.4 / 38.7	30.0 / 40.7 / 35.3
Solvent	43.31	41.96
Number of TLS groups	2	2

Statistics for the highest-resolution shell are shown in parentheses.

**Fig 1:**
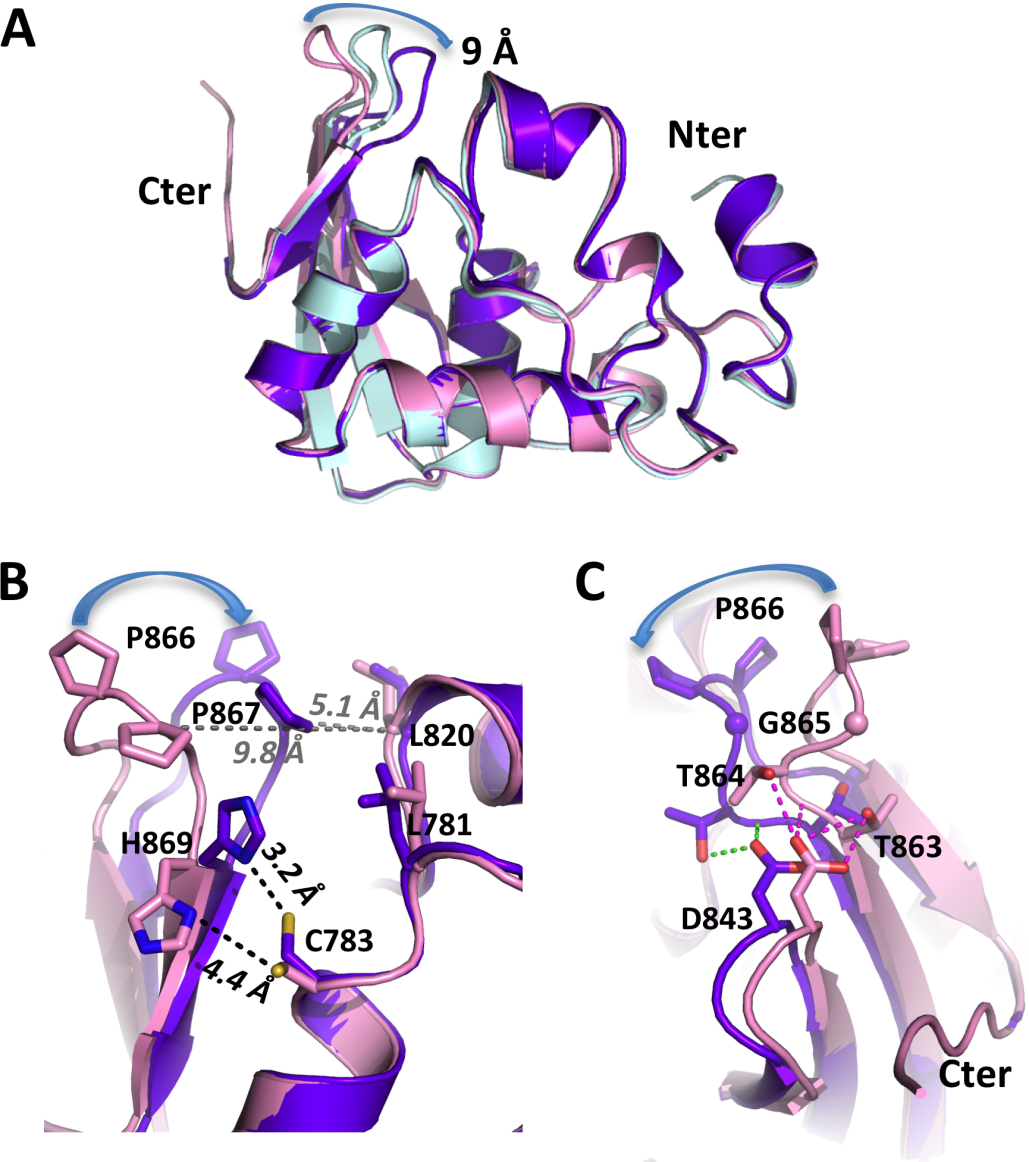
Crystal structure of TYMV PRO/DUB mutant ΔC5. (A) Superposition of PRO/DUB wild type (in pink) and ΔC5 ('A' and 'B', in light cyan and purple-blue, respectively). The displacement of loop 864–868 (865-GPP-867 flap) is indicated with a blue arrow following P866. (B) Superposition of the two most distant conformations (wild type and ΔC5 'B' conformation) seen from the P' side of the catalytic cleft. Residues forming the hydrophobic zipper, as well as the catalytic dyad C783-H869, are displayed as sticks and labeled. The distances between the Sγ of C783 and the Nδ1 of H869 are indicated in black and the distances between the Cαs of P867 and L820 in gray. The displacement of P866 in the zipper is indicated by a blue arrow as in panel (A). (C) Same superposition as in (B) seen from the other side of the GPP flap. Note that D843 follows the movement of the neighbouring GPP loop but that some of its interactions with the base of the loop break.

The ΔC5 crystal contains two molecules per asymmetric unit, hereafter referred to as molecules 'A' and 'B'. Molecule 'B' is slightly less ordered as revealed by a 10 Å^2^ higher average B-factor (Table 1, S2A Fig). Clear electron density is present for residues 730–874 of molecule 'A', and 732–874 of molecule 'B'. Both molecules have their C-termini far from the catalytic sites of other molecules in the crystal packing. We thus have two independent views of PRO/DUB not engaged as a substrate in a PRO:PRO complex. Superimposition of molecules 'A' and 'B' of the ΔC5 mutant with the WT (engaged) PRO/DUB (Fig 1A) shows that PRO/DUB retains the same conformation in all three environments, with the exception of loop 864–868. This loop was strongly constrained in the previous structure due to the cleaved PRO molecule's loop being bound by the cleaving PRO molecule. It is now released in both molecules of the mutant protein ΔC5, and displays high mobility in this disengaged form. In molecule 'B', Pro866 at the tip of the loop (Fig 1B) moves by 9 Å to a position overhanging the catalytic cleft. This shift is less pronounced in molecule 'A', and the loop therefore reaches an intermediate position. Of note, temperature factors of the loop jump up in molecule 'A', indicating higher dynamic disorder in this intermediate conformation, and indeed are above those of molecule 'B' in this region (S2A Fig).

In fact, in molecule 'B', the mobility of this loop leads to the closure of a hydrophobic zipper between Pro866 and Pro867 on one side, and Leu820 and Leu781 across the catalytic cleft on the other side (Fig 1B). His869 and Cys783 also come significantly closer together. Presence of unidentified atoms (S3 Fig), possibly covalent adducts with DTT or sulfur compounds present in our cryosolution (see Materials and Methods), complicated the unambiguous modeling of the Cys783 side chain. However, it is clear that, in this closed conformation, His869 is now close enough to abstract a proton from Cys783. The large movement of the 865-GPP-867 motif observed in molecule 'B' as compared to the WT engaged PRO is due to a distinct flexing of the tip of the beta sheet into which it is inserted. This flexing extends to the neighboring 842–845 loop and, particularly, to the Asp843 at its tip that follows loop 864–868 (Fig 1C). In contrast, the conserved GPP motif itself, with its two *cis*-prolines, changes very little, although Gly865 shifts slightly, moving in the Ramachandran plot of molecule 'B' to a region that is disallowed to non-glycine residues.

In summary, the crystal structure of ΔC5 indicates that, while TYMV PRO/DUB is a rigid molecule, it harbours a very mobile loop, 864-TGPPS-868, that can shift the GPP flap at its tip with its atypical double *cis*-proline, from an "open" state where it can be recognized by another PRO molecule [21], to two other states: a "closed" one where it forms half of a hydrophobic zipper, and an "intermediate" one, halfway in between. The closed zipper constricts the P side of the catalytic cleft and also brings the catalytic His869 closer to the catalytic Cys783.

### Molecular dynamics simulations confirm the distinct GPP flap mobility and a link to active site organization

**Fig 2:**
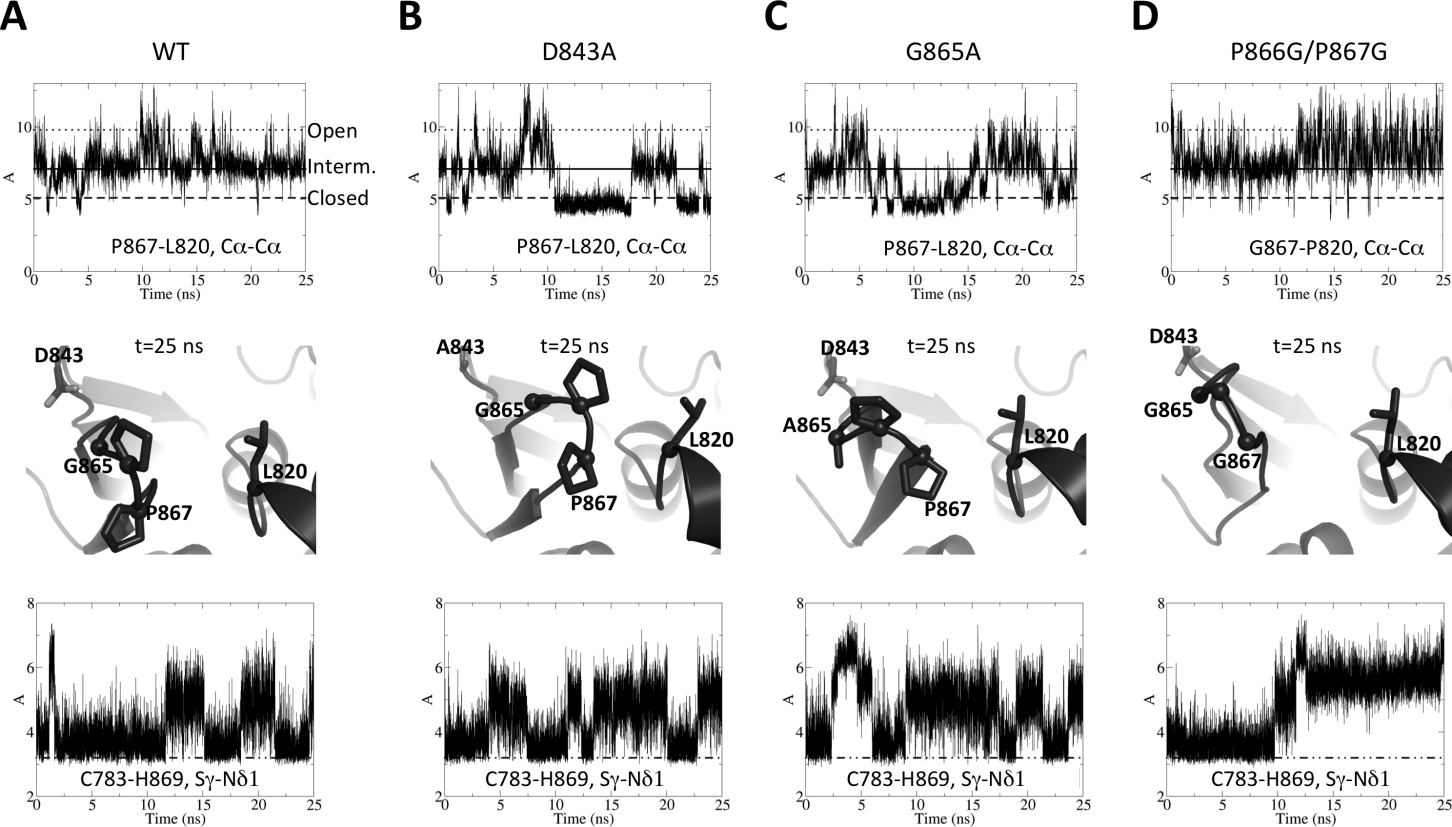
Molecular dynamics simulations for TYMV PRO/DUB and structure-based flap mutants. 25-ns simulations were performed in identical conditions for wild type PRO/DUB (A) and mutants D843A (B), G865A (C) and P866G/P867G (D). Top, evolution along the simulations of the distance between Cα carbons of P867 and L820. Middle, snapshot taken from the end of each simulation (t = 25 ns), that shows a representative view of the GPP flap for all systems. Mutated residues' side chains are displayed as sticks, as well as L820. For L820 and for residues that are glycines or were mutated to glycines, Cα carbons are also shown as spheres. For clarity, hydrogens added for the simulations are omitted. Bottom, evolution along the simulations of the distance between the Sγ of C783 and the Nδ1 of H869. The dotted-and-broken line signals a 3.2 Å value below which the activating hydrogen bond between H869 and C783 is formed. (A) The three P867-L820 distances found in the crystal structures are labeled "Open" (dotted line, for a molecule taken from the PRO:PRO complex, the conformation from which the simulations were started), "Interm." (solid line, value found in the ΔC5 'A' conformation) and "Closed" (broken line, value found in the ΔC5 'B' conformation).

Since we now had three structures with the GPP flap in three different conformations and a suspected connection to active site organization, we performed molecular dynamics simulations to scrutinize the dynamics of these two regions and an eventual link between them. We used as starting model the previously published structure of wild type full-length TYMV PRO/DUB, with the GPP flap in open conformation [21]. Relieved from the PRO:PRO interaction in that former crystal structure, the flap quickly closes to a conformation superimposable with the 'A' molecules of the new structures, *i*.*e*. the intermediate state. Monitoring the closure of the flap by the distance between Cαs of P867 and L820 (Fig 1B) clearly shows that the flap then oscillates around this central conformation, but intermittently either closes to the other side of the cleft or goes back to the more open conformation (Fig 2A, top, the corresponding distances in the crystal structures are indicated as "open", "interm." and "closed", respectively). These results confirm that the new structures complete the range of conformations accessible to the GPP flap and further indicate that the 'A' molecule best represents the PRO/DUB conformation when it neither is engaged as a substrate nor acts as a peptidase (Fig 2A, middle). Interestingly, concomitant with the initial closure of the flap, H869 comes sufficiently close to C783 that the latter realigns and the two side chains form a hydrogen bond. This bond is formed in an orientation of side chains that is identical to that in other OTU DUBs (see Discussion). However the imidazole ring of H869 is still unengaged by a third catalytic residue and remains loose, frequently breaking the H-bond to C783 (Fig 2A, bottom). These events do not correlate with the mobility of the GPP flap around the intermediate, equilibrium position. These results indicate that the organization of the catalytic site is also brought closer to the classical OTU DUB's by flap closure to the intermediate position, but is not further ordered in the closed flap conformation.

### Structure-based mutations in the mobile loop affect DUB enzymatic activity *in vitro*

In order to assess the role of the 864-TGPPS-868 loop in the dual activities of the TYMV PRO/DUB domain, we next designed structure-guided mutants. On the one hand, we prevented its ability to zip up against Leu820 and Leu781 by replacing the bulky, rigid double *cis*-proline by two glycines (P866G/P867G mutant). On the other hand, we kept the zipper intact but tried to interfere with loop mobility: First with a G865A mutant disfavouring complete loop closure, since its closed state involves a conformation of residue 865 disfavoured to non-glycine residues (see above); Second with a D843A mutant that should increase flexibility of the loop by disrupting the hydrogen bonds tethering it to the nearby strand (Fig 1C).

Molecular dynamics simulations of these mutants, performed in the same conditions as for the wild type, show that the molecular effects of these mutations are globally as expected, but also offer new insights into the link between flap movement and active site reorganization. Mutants P866G/P867G, G865A and D843A all significantly alter the dynamics of the GPP flap. First off, it becomes more mobile than in the wild type (S2B Fig). However, it does so in very different ways: In mutant D843A (Fig 2B) the loop harboring A843 does not act anymore as a spring to the GPP flap. As a result, the flap remains closed much longer, but also at times opens wider, than in the wild type (Fig 2B, top). At first sight G865A displays a similar behavior (Fig 2C, top), somewhat surprisingly since the mutation was designed to disfavor closure of the flap. However, examination of the trajectory shows that the constraint imposed by A865 leads to a twist of the flap. Only P867 comes into contact with L820 even in the most closed conformation, leaving the hydrophobic zipper half open (Fig 2C, middle), in contrast to the D843A closed conformation (Fig 2B, middle) that is identical to the crystallographic closed flap conformation. The most disruptive mutant is P866G/P867G (Fig 2D). Although the loop initially relaxes into the intermediate position, as in the wild type and the two other mutants, it is more disordered and, strikingly, in the second half of the simulation shifts to a position that is on average in an "open" rather than "intermediate" conformation (Fig 2D, top and middle). Interestingly, this opening correlates with loss of the H-bond between H869 and C783 (Fig 2D, bottom), leading to an inactive conformation of the catalytic site. These results further confirm the connection between loop closure and active site organization. In contrast, the catalytic dyad's dynamics are much less affected in the two other mutants G865A and D843A (Fig 2B and 2C, bottom).

To address the effect of these changes in flap dynamics and active site organization on the DUB activity *in vitro*, these flap-interfering mutations were first introduced into the recombinant His-tagged PRO/DUB domain of TYMV, and mutated proteins were expressed in *E*. *coli* and purified. The enzymatic DUB activity of the mutants was then measured *in vitro* using the general DUB substrate ubiquitin-7-amino-4-methylcoumarin (Ub-AMC) and assessing release of the fluorescent AMC moiety, as previously described [21, 32]. As controls, we also assessed point mutants (I847A and I847D) of the distant ubiquitin-binding patch that we previously showed to be significantly impaired in DUB activity [21]; the WT enzyme was assayed in parallel to each mutant to have a 100% activity control.

The results obtained (Table 2) indicate that the three loop-targeting mutants display impaired DUB activity *in vitro*. Preventing full loop closure (G865A), or releasing it from its nearby strand (D843A), resulted in significantly decreased activity, with about 30% residual activity, similar to that of the I847A mutant measured in the same conditions. The most drastic effect was observed for the mutant P866G/P867G, whose loop can no longer zip up against Leu820 and Leu781 and tends to be more open than the wild type, while its catalytic dyad is even more disordered. With 3.5% activity, this mutant falls in the range of I847D, the most severely impaired DUB distant patch mutant. Altogether, these results indicate that affecting the GPP flap or its mobility impacts DUB activity *in vitro*.

**Table 2:** *In vitro* DUB activity of structure-guided mutants of TYMV PRO/DUB.

PRO/DUB proteins	*K*_app_ (% of WT)
WT	100
P866G/P867G	3.5 ± 0.4
G865A	32 ± 2
D843A	28 ± 2
I847A	22 ± 4
I847D	1.7 ± 0.1

DUB activity of recombinant PRO (wild-type and structure-guided mutants) was measured by a fluorescence assay using Ub-AMC as substrate. *K*_app_ was determined according to the equation V / [E] = *K*_app_ [S], where V is the initial velocity calculated from the kinetic data, and [E] and [S] are the corresponding enzyme and substrate concentrations. To overcome the observed variation in *K*_app_ values according to the batch of Ub-AMC used, values of mutant proteins were expressed as the percentage of that of WT protein measured in the same experiment. Such variations may possibly account for the apparently 3-fold lower activities we previously reported for the I847A and I847D mutants [21].

### Structure of PRO/DUB I847A confirms that 864-TGPPS-868 loop zippering is a property of TYMV PRO/DUB

**Fig 3:**
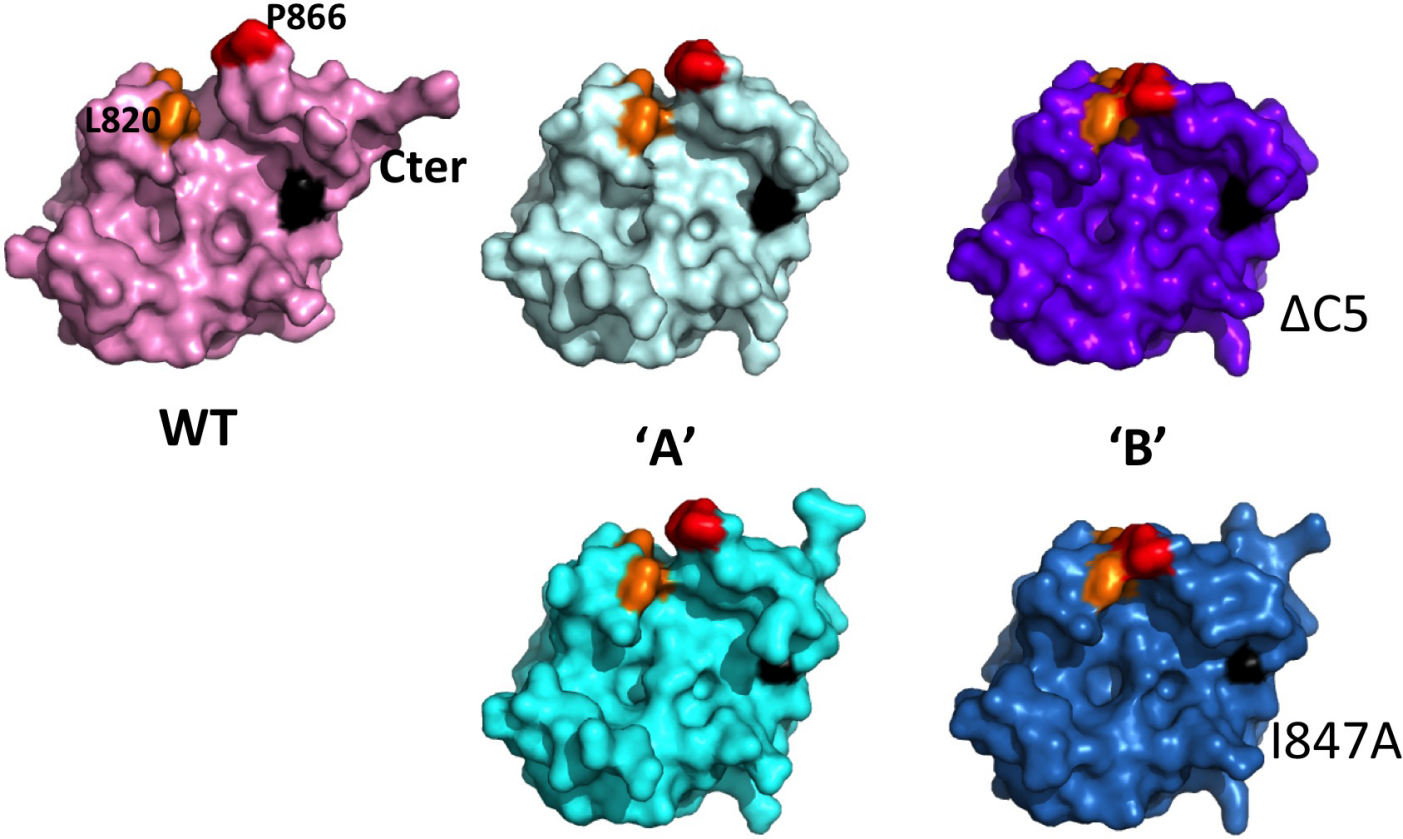
Crystal structure of TYMV PRO/DUB mutant I847A and comparison of all available molecules. The five molecules are displayed in surface representations for PRO/DUB wild type (top left) and molecules 'A' (middle) and 'B' (right) of ΔC5 (top) and I847A (bottom, in cyan and sky-blue, respectively), all in the same orientation. The C-terminus is indicated, and P866 and L820 are labeled in the WT. The hydrophobic zipper is in red (P866, P867) and orange (L781, L820). Residue 847 is in black.

Given the two similar classes (mild or severe) of DUB activity impairment, whether in loop mutants or distant patch mutants, we also examined the structure of the TYMV PRO/DUB I847A point mutant (hereafter 'I847A'), crystals of which were actually obtained first, and from which we seeded the ΔC5 crystals [38]. The seeding procedure led to the same crystal form, and the I847A crystal is essentially identical to the ΔC5 crystal. The only difference in packing between the ΔC5 and I847A crystals is a slight shift of molecule 'B' with respect to molecule 'A' (S4 Fig). Residues 730–877 are ordered in I847A molecule 'A' and residues 732–876 in I847A molecule 'B'. The two I847A molecules appear superimposable to their counterparts in the ΔC5 crystal, with root-mean-square deviation of 0.09 Å for the 143 Cα carbons present and ordered in both 'A' molecules, and 0.23 Å for 141 Cα carbons present and ordered in both 'B' molecules. Importantly, loops 864–868 thus occupy the same positions in the I847A and ΔC5 crystals, *i*.*e*. intermediate in 'A' and zipped up in 'B' (Fig 3).

The fact that the two mutants I847A and ΔC5 display identical conformations in the same crystal packing environments shows that the mutations themselves have altered neither the overall structure of the molecule, nor the conformation of loop 864–868. Thus, the mobility and zippering of loop 864–868, and the associated changes in the active site conformations as compared to the WT PRO:PRO complex, are properties of the PRO/DUB framework and reflect available conformations of this mobile loop. Additionally, the structure of the I847A mutant confirms that the mutation indeed reduces the hydrophobic surface of the distant patch (black patches in Fig 3).

### *In vivo* assays reveal that mutants are specifically affected in DUB but not PRO activity

To determine whether the mutations described above also impact the activity of the protein *in vivo*, and whether they affect DUB activity, PRO activity, or both, we next monitored the activities of TYMV 98K, i.e. the mature viral protein product encompassing the PRO/DUB domain [31], using an Arabidopsis protoplast transient expression system.

Because the TYMV 66K protein encompassing the viral RNA-dependent RNA polymerase is the sole identified substrate of the 98K DUB activity to date [32], we addressed the capability of 98K to remove poly-Ub chains from 66K protein. To this end, the levels of 66K-Ub conjugates were assessed by co-expressing 66K in Arabidopsis cells in the presence of myc_2_-Ub [a *myc*-tagged version of Ub [28]], together with WT 98K or one of the various mutant versions of 98K summarized in Table 2. The catalytically inactive 98K-C783S was used as a control.

**Fig 4:**
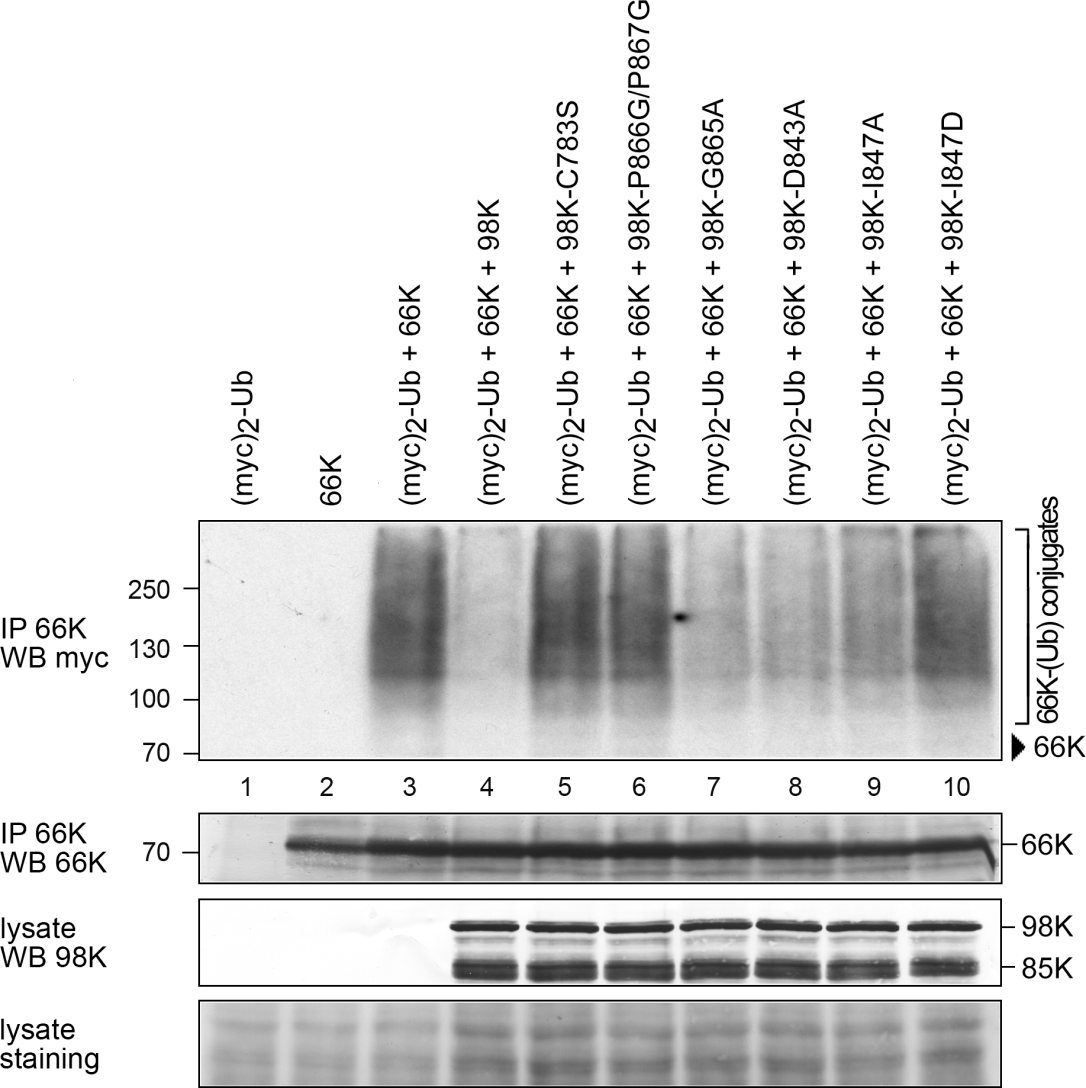
Impact of WT or mutant 98K proteins on ubiquitylation of 66K protein. Arabidopsis protoplasts were transfected with pΩ-66K, pΩ-myc_2_-Ub, alone or together with pΩ-98K, pΩ-98K-C783S, pΩ-98K-P866G/P867G, pΩ-98K-G865A, pΩ-98K-D843A, pΩ-98K-I847A or pΩ-98K-I847D as indicated. Cells were collected 48 hpt, and samples were immunoprecipitated under denaturing conditions with anti-66K antibody. Samples were then normalized according to the amount of 66K and subjected to immunoblotting with anti-*myc* antibody (IP 66K / WB myc). The positions of molecular mass markers are indicated on the left, and that of viral proteins on the right. Arrowhead: Position of 66K. The amount of 66K present in the immunoprecipitates was determined by immunoblotting with anti-66K antibody (IP 66K / WB 66K), and the amount of 98K derivatives present in the cell lysates was determined by immunoblotting with anti-98K antibody prior to immunoprecipitation (lysate / WB 98K). The shorter 85K protein corresponds to a nonspecific degradation product of 98K occurring during sample extraction and protein analysis [31]. Ponceau staining of the membrane (lysate staining) indicates protein loading in the whole cell lysate samples. Each mutant was analyzed in two independent experiments.

As previously reported [28, 32], immunoprecipitation of 66K under denaturing conditions followed by immunoblot analysis with anti-*myc* antibodies readily allows the detection of 66K-Ub conjugates (Fig 4, lane 3). Consistent with previous reports, the amount of ubiquitylated 66K was drastically reduced when 66K was coexpressed in the presence of WT 98K (Fig 4, lane 4), but was unaffected by expression of the catalytically inactive 98K-C783S protein used as a control (Fig 4, lane 5). Mutants with a mild phenotype in the DUB *in vitro* assay (around 30%, see Table 2) all appeared to retain a significant amount of DUB activity *in vivo*, as shown by the partial disappearance of 66K-Ub conjugates. This is true whether we consider loop-affecting mutants (G865A and D843A, lanes 7–8) or distant patch mutant (I847A, lane 9). In contrast, mutants in the hydrophobic zipper (P866G/P867G), and the mutant introducing a charge in the distant patch (I847D), which were the most severely defective in the *in vitro* DUB assay (less than 5%, see Table 2), both displayed a drastic decrease in their DUB activity *in vivo*, as shown by the high accumulation of 66K-Ub conjugates in transfected cells (lanes 6 and 10).

Altogether, these results reveal good agreement between *in vitro* and *in vivo* assessments of DUB activity, and demonstrate that, either mutating a distant patch presumed to interact with Ub, or interfering with GPP flap mobility and zippering, both severely impair the DUB activity of TYMV PRO/DUB *in vivo*.

We next sought to evaluate the impact of the above-described mutations on the PRO activity on the viral polyprotein transiently expressed in Arabidopsis cells. TYMV PRO activity was previously reported to be involved in the processing of the 206K replication protein precursor at two distinct sites, located between its PRO, helicase (HEL) and polymerase (POL) functional domains [31] (S1 Fig). Cleavage at the HEL↓POL site (position 1259/1260 of 206K precursor) first releases the 66K protein encompassing the POL domain, and is absolutely required to promote viral replication. Cleavage at the PRO↓HEL site (residues 879/880) releases the mature proteins 98K and 42K, and appears to contribute to the fine regulation of viral RNA replication [31].

**Fig 5:**
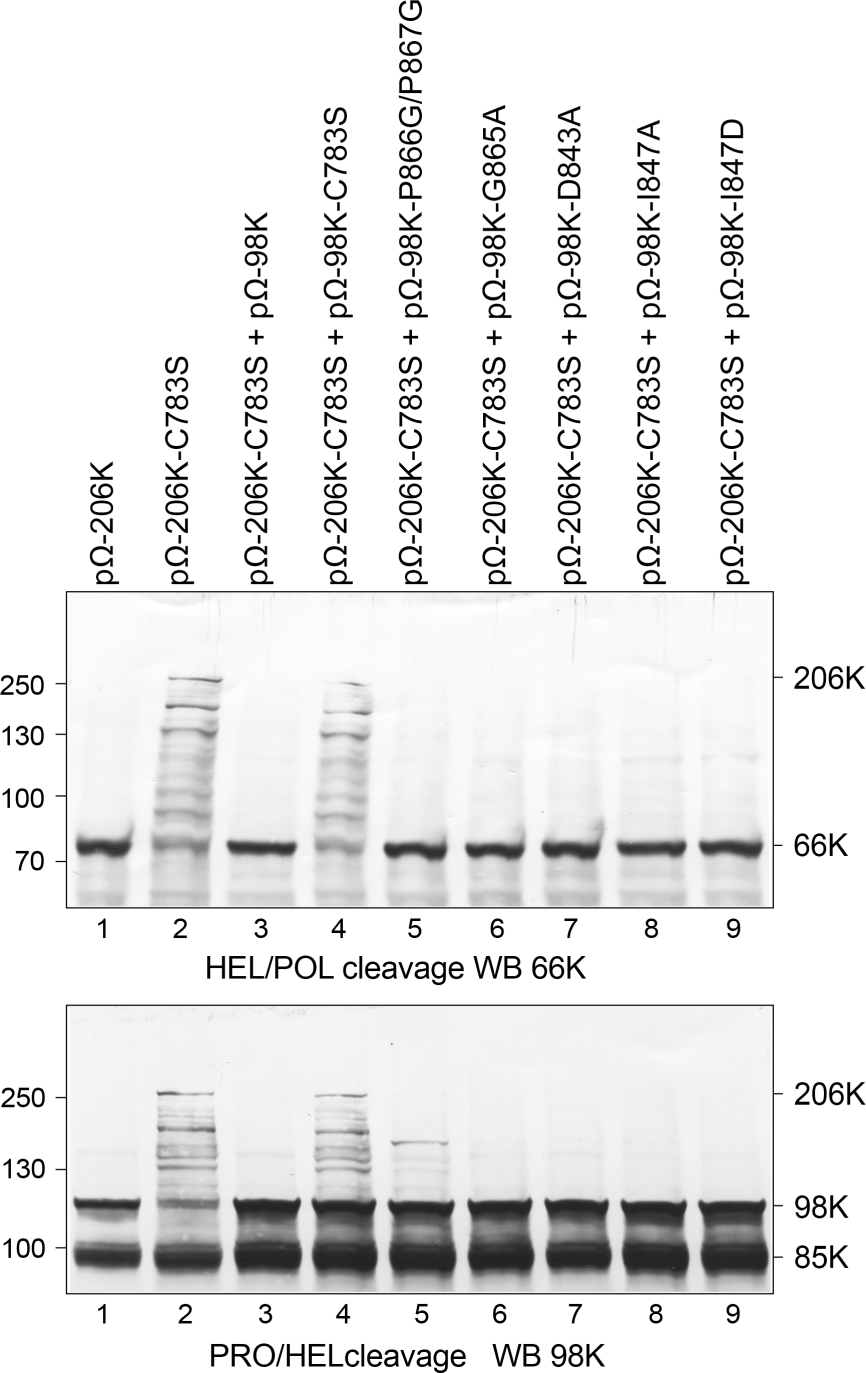
Impact of WT or mutant 98K proteins on processing of precursor protein 206K. Arabidopsis protoplasts were transfected with the expression vectors indicated, alone or in combination. Cells were collected 48 hpt and total protein extracts were subjected to 8% SDS-PAGE and immunoblot analysis with anti-66K (top) or anti-98K (bottom). The positions of molecular mass markers are indicated on the left, and that of viral proteins on the right. Protein 206K corresponds to the substrate protein, 66K and 98K to its mature cleavage products. In the lower panel, the mature 98K product resulting from cleavage of 206K is obscured by the 98K produced in *trans* from pΩ-98K. The shorter 85K protein corresponds to a nonspecific degradation product of 98K occurring during sample extraction and protein analysis [31]. Each mutant was analyzed in two independent experiments.

As 98K can process both cleavage sites *in trans* [31], we used the approach previously described to assess the PRO activity of WT or mutated 98K. For this purpose, the expression plasmid pΩ-206K-C783S (encoding the 206K precursor protein lacking protease activity to prevent any self-cleavage, but retaining the two cleavage sites to serve as a substrate) and WT or mutant versions of pΩ-98K (encoding the viral 98K protein to serve as a protease) were transfected into Arabidopsis protoplasts. Processing at the HEL↓POL and PRO↓HEL cleavage sites of the 206K substrate was assayed by immunoblotting of the corresponding protein samples using anti-66K and anti-98K antibodies, respectively. As shown in upper panel of Fig 5, lane 3, the WT 98K protein processed the HEL↓POL cleavage site efficiently *in trans*, as evidenced by the disappearance of the 206K precursor and the immunodetection of the mature 66K product. Note that the mature 98K product resulting from cleavage of 206K at the PRO↓HEL site (lower panel) is obscured by the 98K produced in *trans* from pΩ-98K, but that cleavage activity is evidenced by the disappearance of the 206K precursor. As expected, cleavage of the substrate was inhibited upon mutation of the catalytic C783 residue (lane 4). Interestingly, we observed that all of the mutants assayed—including P866G/P867G and I847D (lanes 5 and 9)—retained the capability to process both the HEL↓POL and PRO↓HEL cleavage sites, although traces of intermediate cleavage products were occasionally detected.

These experiments therefore demonstrate that the PRO and DUB activities of 98K can be specifically decoupled. This can be achieved through two distinct approaches: first, through mutants affecting the I847 distant patch, which were designed to interfere with recognition of the Ub surface [21]; second, through mutants designed to affect GPP flap mobility, especially zippering, highlighting the importance of this flap in DUB activity.

Such mutants now provide very powerful tools to decouple the dual PRO and DUB activities of TYMV PRO/DUB, allowing specific assessment of the function of DUB activity in infected cells.

### Altering TYMV-encoded DUB enzymatic activity impairs viral RNA replication in Arabidopsis single cells

We previously reported that abolishing the DUB activity of TYMV PRO/DUB had a significant impact on the ability of TYMV to replicate in infected cells, causing a ~ 3-fold reduction in infectivity [32]. However, because the only available DUB mutant at that time was the catalytic site C783S mutant that debilitates both PRO and DUB activities, and because polyprotein processing of the precursor 206K at the HEL↓POL junction is an absolute prerequisite for initiating viral replication [31], those experiments required a sophisticated design, based on the *trans*-complementation of a polymerase deletion mutant with the mature cleaved product [32], in order to functionally dissociate the requirement for polyprotein processing from the putative contribution of DUB activity to viral infectivity. While demonstrating the importance of the DUB activity for viral replication, this approach suffered from possible flaws linked to overexpression of the polymerase *in trans*, and confined the analysis of viral infection to the level of single transfected cells.

**Fig 6:**
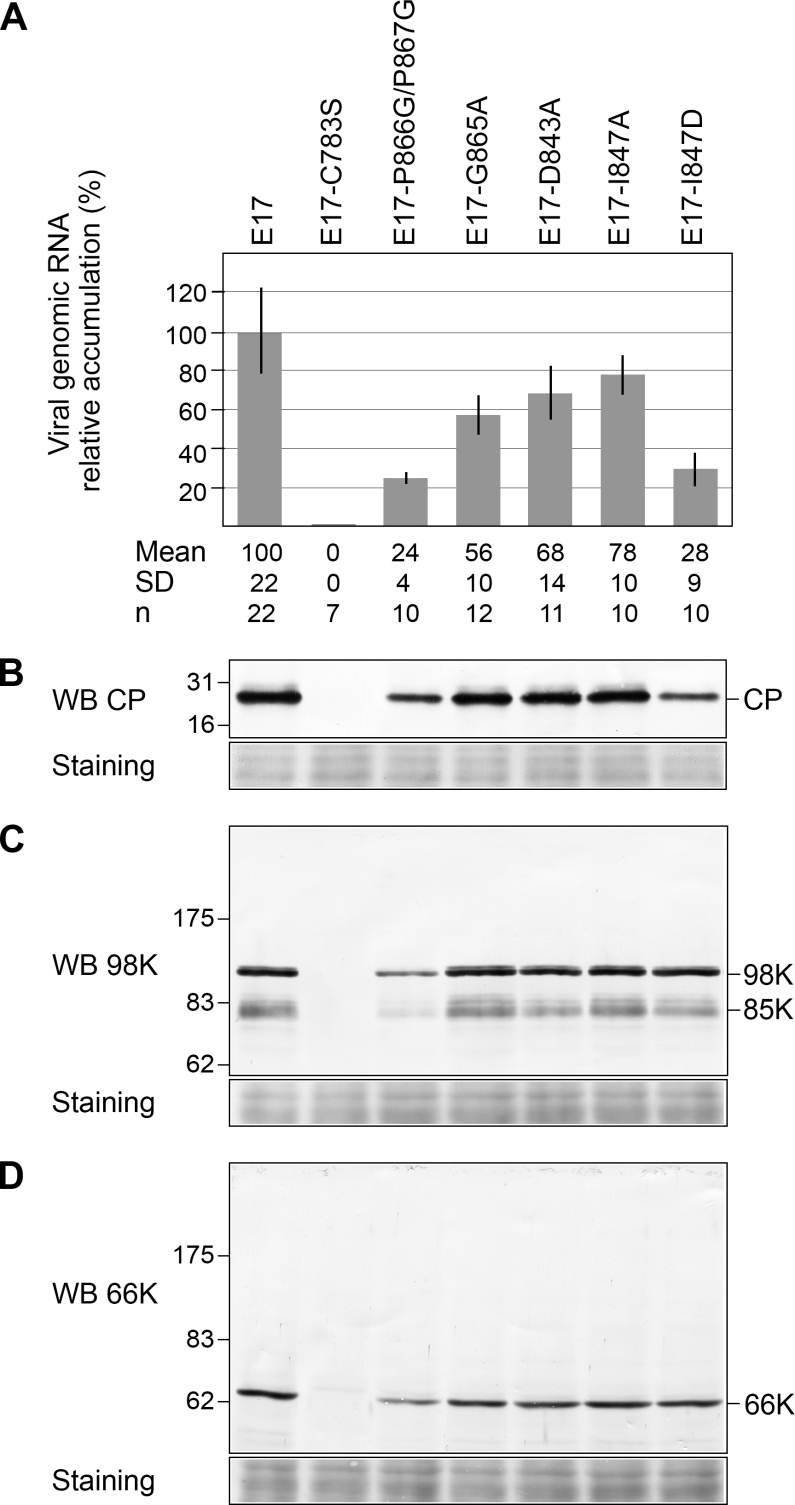
Impact of DUB activity on viral infectivity in Arabidopsis protoplasts. Arabidopsis protoplasts were transfected with *in vitro* transcripts as indicated and cells were harvested 48 hpt. (A) The ability of the transcripts to replicate was assessed by extracting total RNAs and quantifying viral genomic RNA by RTqPCR. Viral RNA levels were calibrated and normalized to *EF1αανγПΔФ2* and *PDF2* reference genes RNAs. The relative accumulation of viral mutant RNAs as compared to the WT TYMV RNA is represented as the mean +/- SD. Mean and SD values, as well as the number of samples (n) analyzed in at least two independent experiments are indicated below panel (A). (B) The ability of the transcripts to replicate was assessed by immunoblotting with anti-CP antibodies. (C) and (D) Detection of mature viral proteins produced during infection. The same samples as in panel (B) were analyzed by immunoblotting with anti-98K (C) and anti-66K (D) antibodies. Ponceau staining of the membrane (staining) indicates protein loading. Each mutant was analyzed in two independent experiments.

Having now designed mutants of TYMV PRO/DUB with various levels of DUB activity impairment but retaining PRO activity, we sought to readdress this question by introducing these new mutations into our TYMV reverse genetics system. For this purpose, mutations P866G/P867G, G865A, D843A, I847A and I847D were introduced into the plasmid E17, which contains a full-length copy of the TYMV genome, and from which infectious viral transcripts can be obtained [27]. Plasmid E17-C783S, mutated in the PRO/DUB catalytic cysteine, was used as a control. Equal amounts of *in vitro* transcripts were transfected into Arabidopsis protoplasts, and viral infectivity was assessed by detecting viral genomic RNA progeny by RT-qPCR (Fig 6A) or capsid protein (CP) by Western blotting (Fig 6B), as the latter is dependent on viral replication for its expression from a subgenomic RNA.

Mutation of the catalytic cysteine completely abolished accumulation of both viral RNA progeny and CP (Fig 6A and 6B)—a consequence of impaired HEL↓POL cleavage, consistent with previous reports [29, 31]. In contrast, all other mutants retained the capability to replicate, albeit at reduced levels (Fig 6A and 6B). Strikingly, viral mutants P866G/P867G and I847D, which displayed the lowest levels of DUB activity *in vitro* and *in vivo* (Table 2, and Fig 4) had the greatest impact on infectivity, being able to replicate at levels of 24% and 28%, respectively, of WT viral RNA (Fig 6A). Such results are thus in perfect agreement with our previous data [32], and confirm that debilitation of the DUB activity leads to a 3- to 4- fold decrease in viral RNA replication in Arabidopsis cells.

Mutants I847A, G865A and D843A with *in vitro* DUB activities of ~ 30% of that of WT enzyme were found to replicate at levels of 60–80% that of WT viral RNA, indicating that even mild impairment of DUB activity has a detectable impact on viral replication.

Western blot analyses using anti-98K and anti-66K antibodies (Fig 6C and 6D) revealed that only fully mature viral proteins were detected in infected cells, thus confirming the fully functional PRO activity of the PRO/DUB mutants during infection, and ruling out a possible impact of partially impaired cleavage of 206K on viral infectivity.

### Both the DUB enzymatic activity and the conformation of the GPP flap influence viral RNA accumulation and symptoms appearance *in planta*

To determine whether the viral mutants affected in DUB activity retained infectivity *in planta*, *Arabidopsis thaliana* plants were inoculated with equal quantities of the corresponding *in vitro* transcripts. Viral infectivity and systemic movement were then assessed by observing the appearance of viral symptoms in the inoculated leaves and in leaves distant from the point of inoculation (systemic leaves), as well as quantifying the viral genomic RNA progeny by RTqPCR.

**Fig 7:**
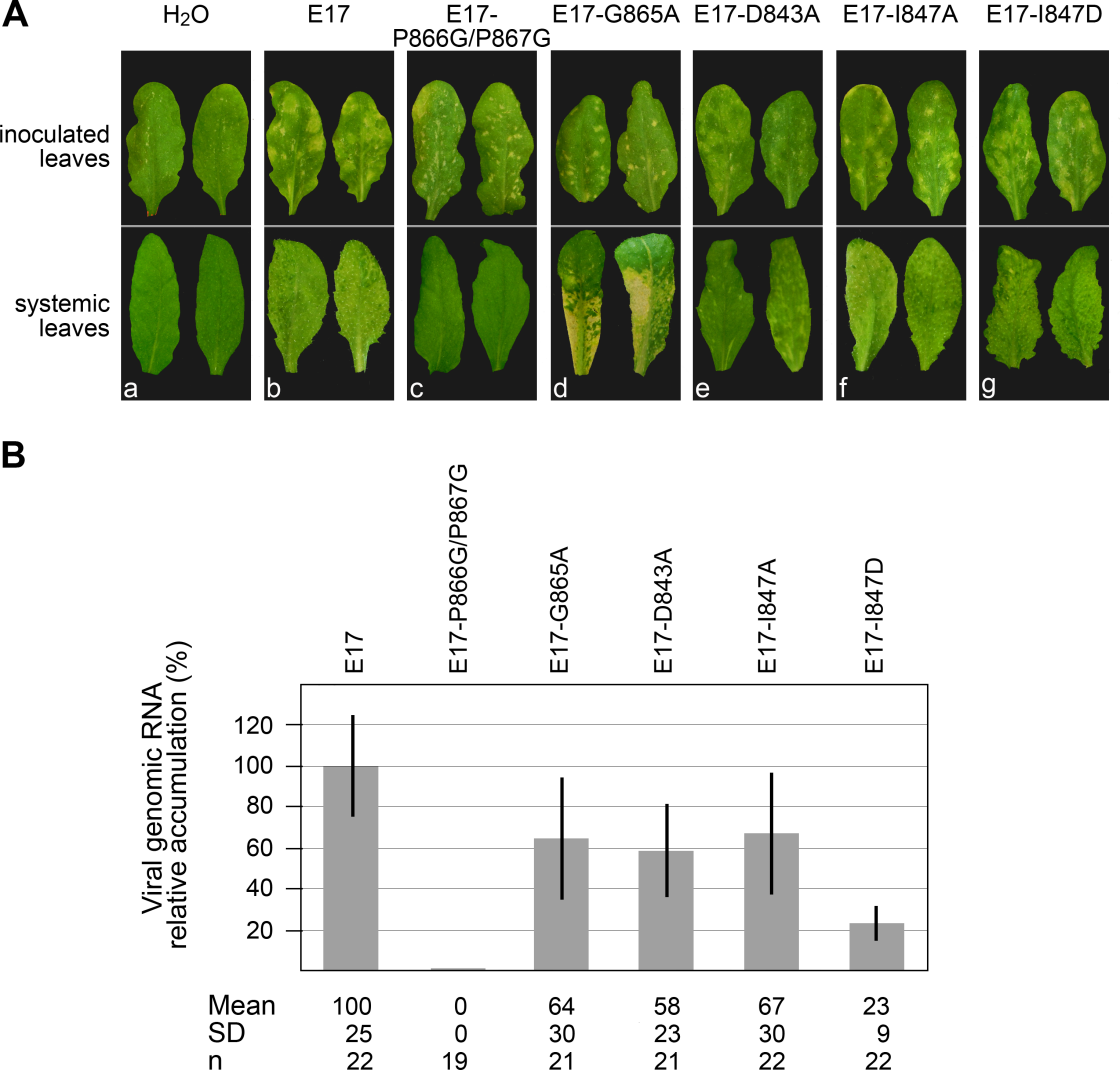
Impact of DUB activity and mutations of the GPP flap on viral infectivity and symptoms appearance in planta. Arabidopsis plants were mechanically inoculated with water, or *in vitro* transcripts as indicated. (A) Symptoms development were monitored and representative pictures of the symptoms observed on the inoculated leaves (upper panel) at 14 dpi and on the systemic leaves (lower panel) at 17 dpi are shown for each inoculum. The same type and severity of symptoms were consistently observed in at least four independent experiments involving n ≥ 26 plants. (B) The ability of the transcripts to multiply in systemic leaves was assessed by extracting total RNAs and quantifying viral genomic RNA by RTqPCR at 17 dpi. Viral RNA levels were calibrated and normalized to *EF1α* and *18S* rRNA reference genes RNAs. The relative accumulation of viral mutant RNAs as compared to the WT TYMV RNA is represented as the mean +/- SD. Mean and SD values, as well as the number of samples (n) analyzed in at least three independent experiments are indicated below panel (B).

Arabidopsis plants inoculated with WT transcripts E17 displayed typical symptoms of TYMV infection (Fig 7A, panel b), i.e. chlorotic local lesions appearing on the inoculated leaves 10–12 days post inoculation (dpi), as well as leaf distortion, chlorosis and mosaic symptoms appearing 2–3 days later on the systemic leaves.

Although all mutants appeared to be able to multiply based on the appearance of local lesions on the inoculated leaves, they strikingly differed in the type of symptoms they were causing. While mutations in the distant patch involved in Ub recognition, I847A and I847D, led to the appearance of symptoms (Fig 7A, panels f and g) which were comparable to those caused by WT transcripts, the symptoms caused by the mutants affected in the GPP flap looked notably different.

In particular, E17-P866G/P867G induced the appearance of necrotic local lesions on the inoculated leaves (Fig 7A, panel c), which are reminiscent of those occurring during hypersensitive reactions (HR), a process corresponding to an exacerbated immunity reaction of the host [39,40]. Those lesions appeared 1–2 days earlier than those caused by a WT infection, were self-limiting, and were found to restrict systemic viral movement, as evidenced by the absence of symptoms in systemic leaves, as well as the absence of viral RNA progeny (Fig 7B). The E17-G865A mutant also displayed a peculiar phenotype (Fig 7A, panel d), as the small local lesions on the inoculated leaves were originally chlorotic and did not appear to restrict systemic viral movement, leading to severe chlorotic symptoms in systemic leaves. However, around 15–17 dpi, the chlorotic areas then became necrotic, leading to tissue desiccation, and to a systemic necrosis phenotype, eventually leading to leaf loss. On the other hand, the symptoms induced by E17-D843A (Fig 7A, panel e) were milder than those caused by a WT infection, the local lesions being less chlorotic, as were the mosaic symptoms appearing on the systemic leaves.

From these observations, it thus appears that the differences in symptoms severity among the various mutants do not correlate with the DUB activity *per se*, as the I847A and I847D mutants caused symptoms similar to WT infection, despite having drastically different levels of DUB enzymatic activity, whereas mutants G865A and D843A caused extremely dissimilar symptoms, despite having comparable levels of DUB enzymatic activity (Table 2 and Fig 4).

Instead, it is of interest to note that symptom severity rather seems to correlate with the conformation adopted by the GPP flap as determined by the molecular dynamics simulations (Fig 2). On one hand, the D843A mutant, whose flap was shown to stabilize in a "closed" conformation, led to milder symptoms. On the other hand, the P866G/P867G and G865A mutants, which both appeared unable to properly close their hydrophobic zipper and remained in an "open" or "half-open" position, led to rapid or delayed necrosis, respectively. Finally, I847A and I847D, which are not mutated in their GPP flap, induced WT symptoms.

When the accumulation of viral RNA progeny was assessed by RTqPCR in the systemic leaves at 17 dpi (Fig 7B), all mutants except E17-P866G/P867G were detected, consistent with the restriction of the latter in the inoculated leaves. Interestingly, the levels of viral RNA accumulation of each mutant *in planta* were in good match with the ones observed in protoplasts (Fig 6A), and correlated with the extent of DUB activity impairment. Thus, mutation I847D had the greatest impact on infectivity, with E17-I847D accumulating to a level of ~ 23% of WT viral RNA, hence demonstrating the importance of DUB activity for viral infectivity in whole plants as in single cells.

Altogether, upon assessment of the infectivity of viral mutants in whole plants, we thus conclude that two superimposed phenomena most likely contribute to the observed variations in viral RNA accumulation and appearance of symptoms: 1) the conformation of the GPP flap, whose opening/closure may impact the severity of symptoms, culminating in the appearance of local necrotic lesions restricting viral infection in the case of the P866G/P867G mutant, 2) the level of DUB activity, which contributes to the extent of viral RNA accumulation very similarly in protoplasts and *in planta*, thus confirming the importance of this enzymatic activity for viral infectivity.

## Discussion

### The GPP flap is mobile, and its closure restores the OTU DUB hydrophobic constriction on the P side of the active site

We previously reported that TYMV PRO/DUB, while evolutionarily an OTU DUB with an acquired processing protease function, displayed an active site that is widely divergent from the canonical active site of related OTU DUBs [21], i.e. it harbors a minimal catalytic site bearing an exposed catalytic dyad instead of a relatively buried triad. We also noted the insertion of a loop, directly upstream of the catalytic His869, with a striking 865-GPP-867 motif that is conserved in *Tymoviridae*-encoded proteins but absent from all other known OTU DUBs. Surprisingly, the two successive prolines in this motif were found to both assume a *cis* conformation. Here, we show that this loop is mobile, and that when left unengaged by an intermolecular interaction, the two-proline flap is much closer to the other side of the catalytic cleft, occasionally closing the gap and zipping up against the opposite Leu820 and Leu781 (Fig 1, Fig 2A).

**Fig 8:**
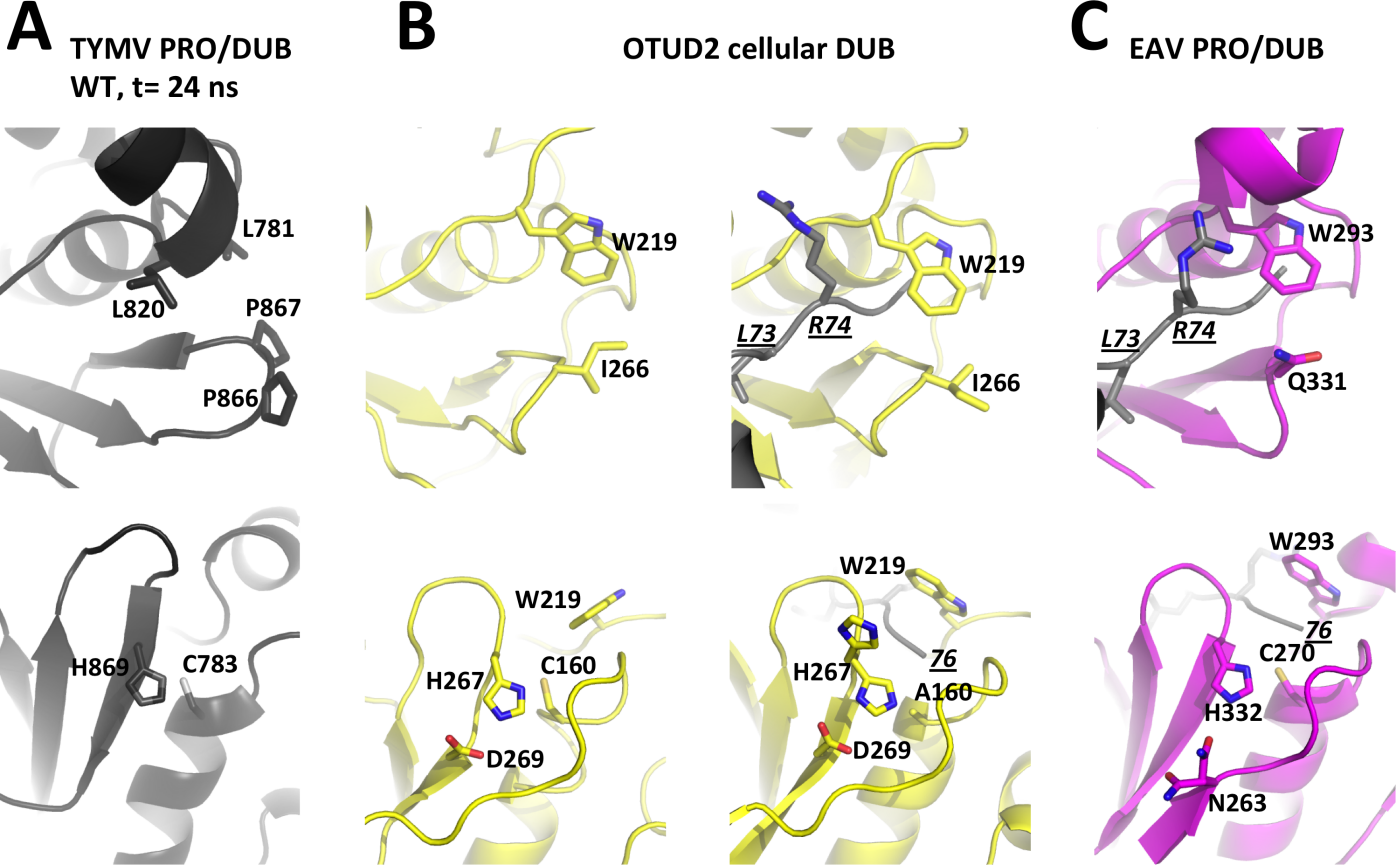
Layouts of the two sides of the active site clefts in viral OTU PRO/DUBs and cellular OTU DUBs. For each enzyme, the top panel is the view from the P side with residues bridging the hydrophobic constriction as sticks and labeled. The bottom panel is the view from the P' side with residues known to contribute to catalysis as sticks and labeled. For these DUBs for which structures are available in both free and Ub-bound forms (see also S5 and S6 Figs), the free enzyme is displayed on the left, and the complex on the right. In complexes, Ub is in grey with its C-terminal residues 73-LRGG-76 as sticks. L73 and R74 are labeled in underlined italics in one view, and the C-terminus is labeled with an italicized and underlined '76' in the other view. (A) TYMV PRO/DUB. A snapshot at t = 24 ns from the molecular dynamics simulation of the wild type molecule was used here. This snapshot represents the typical equilibrium conformation where the GPP flap is in the intermediate position (Fig 2A, top and Fig 8A, top) and the catalytic dyad realigned (Fig 2A, bottom and Fig 8A, bottom). For clarity, hydrogens added for the simulation are omitted. (B) The cellular OTU DUB domain OTUD2 in free (PDB 4BOQ) and Ub-bound (PDB 4BOS) forms. The latter is a catalytic cysteine mutant C160A and its catalytic histidine displays static disorder with alternate canonical and swung out conformations. (C) The arterivirus PRO/DUB in complex with Ub (PDB 4IUM). Despite its divergence from other OTU DUBs, this enzyme displays a canonical OTU DUB active site cleft both on the P and P' sides. The only exception is the third catalytic residue (here N263) that comes from a neighbouring strand.

We compared these features with the general organization and flexibility of OTU DUBs in this region (Fig 8, top row). In addition to the OTU DUBs we previously considered, namely yeast OTU1 and the nairovirus DUBs [21], we also included in this comparison OTU DUB structures that have since been released, including human DUBs of the OTUD subfamily, which are closest to TYMV PRO/DUB (DALI Z-scores [41] of 7 to 8) and one of which (OTUD2) is available in both free and Ub-bound forms [37]. In addition, we also considered the papain-like protease 2 from Equine arteritis virus (EAV), a PRO/DUB encoded by the (+)RNA arteriviruses [22]. The EAV PRO/DUB, while related to other OTU DUBs, is even more divergent than TYMV PRO/DUB from other OTU DUBs, with DALI Z-scores < 5.

These OTU DUBs show conserved features on both the P and P' sides of the catalytic cleft. On the P side, *i*.*e*. the side that binds the C-terminus of the Ub moiety to be cleaved, there is a constricted cleft with a conserved tryptophan overhanging the tunnel through which the Ub C-terminal glycines 75-GG-76 thread (Fig 8B and 8C, top). The tryptophan also connects to the aliphatic part of the residue on the opposite side of the cleft, a residue that is variable among OTU DUBs (I266 in OTUD2 and Q331 in EAV PRO/DUB, Fig 8B and 8C top), and that comes in sequence right before the catalytic histidine. Such a connection appears much less mobile than the TYMV PRO/DUB GPP flap. Indeed, in all available structures of OTU DUBs except one, the P side constriction is present and changes little, either between different DUBs or between free and Ub-bound enzyme. The exception is the structure of OTUD1, where the tryptophan is flipped out and the cleft consequently exposed. This conformation may reflect a state in which the DUB opens transiently to accommodate the isopeptide bond (and in the case of PRO/DUBs, the endopeptide bond) to be cleaved. At any rate, it seems that the TYMV PRO/DUB hydrophobic zipper substitutes for the stable hydrophobic constriction overhanging the C-terminal 75-GG-76 of ubiquitin in the other OTU DUBs. Thus, GPP flap mobility allows seemingly reversible restoration of the canonical OTU DUB physico-chemical layout on the P side of the catalytic cleft.

These comparisons make it clear that the canonical OTU DUB feature of a stable hydrophobic P side constriction is replaced in TYMV PRO/DUB by a removable flap that is seen assuming what can be assigned to three discrete positions ("open", intermediate" and "closed") with the same locked bulge made of two *cis*-prolines. Our mutagenesis data show that interfering with the dynamics between conformations, and especially preventing the closed one, specifically affects DUB activity, but not PRO activity. We thus conclude that the closed conformation is associated with DUB activity, while the more open conformation is relevant to PRO activity.

As the TYMV GPP flap is an insertion not found in other OTU DUBs, which all rely on the canonical aromatic residue to permanently bridge the top of the catalytic cleft above the di-glycine in the Ub C-terminus RLRGG (Fig 8B and 8C), the question of why TYMV evolved such a switch in its PRO/DUB activities will require further exploration.

One possible explanation is that the peculiar flexibility of the TYMV PRO/DUB active site may be tailored to allow a relaxed endoprotease sequence specificity, as, in the intermediate conformation, the active site will still readily accommodate a non-glycine residue at the C-terminus of the cleaved substrate [21]. This is in accordance with the requirements for the two endoprotease substrates of TYMV PRO/DUB, i.e. KLNGA↓ and RLLGS↓ for HEL↓POL and PRO↓HEL junctions, respectively [31], which thus do not match the di-glycine Ub C-terminus. In contrast, other (+)RNA viruses encoding PRO/DUBs evolved endoprotease cleavage sites in their polyproteins that better match the Ub C-terminus. For instance the EAV OTU PRO/DUB putative cleavage site at the nsp2-nsp3 junction is RLIGG↓ [22] and those of coronaviruses (CoV) PRO/DUBs are LXGG↓ at the nsp1-2 and nsp2-3 junctions, and IXGG↓ at the nsp3-4 junction [42,43].

The CoV PRO/DUBs belong to the ubiquitin-specific protease (USP) family [44]—enzymes with different folds than OTU DUBs, but with superimposable catalytic triads [35] (S5 Fig, bottom). Interestingly, they have also evolved insertion of a mobile loop upstream of the catalytic histidine [43] (S5 Fig, top), showing the permissivity to insertions in the same place in the context of a more canonical DUB active site in another DUB family. Although the function of the CoV loop in enzyme regulation and viral infectivity remains to be established, it likely plays a different role from the rigid and conserved TYMV GPP flap, as it is a long and non-conserved flexible loop that contacts the Ub R74 residue rather than its C-terminal di-glycine [43].

Whether viral PRO/DUBs other than those encoded by *Tymoviridae* also make use of such a mobile rigid flap to switch between dual activities awaits further analysis. In any case, the structural and functional data obtained here for TYMV PRO/DUB shed light on the molecular details that allow viruses to achieve diverse functions in the viral infection cycle while maintaining extremely compact genomes.

### Closure of the GPP flap only partially restores a normal papain-like active site

A second consequence of the TYMV GPP flap closing is that the catalytic cysteine and histidine are brought close enough for their side chains to interact, realigning to the normal configuration of OTU DUBs in our molecular dynamics simulations (Fig 2, Fig 8A, bottom). Indeed this is a known form of regulation for cellular DUBs, where a mobile loop may lead to the realignment of the catalytic site residues from an inactive to an active configuration [45]. This regulatory mechanism is best characterized in the cellular DUB USP7, where this mobile loop is accordingly named "switching loop", and there brings the catalytic cysteine back towards the catalytic histidine (S6 Fig).

However, despite this similarity, the TYMV PRO/DUB active site is still an outlier among DUBs (Fig 8, S5 Fig and S6 Fig, bottom rows). Indeed, two elements of the canonical cysteine DUB active site are missing. First, no oxyanion hole is restored in the more closed TYMV PRO/DUB conformations and, consequently, the catalytic cysteine remains unusually exposed on the P' side. Second, the third residue of the papain-like catalytic triad (an aspartate or an asparagine interacting with the catalytic histidine) is also still missing. Consequently, the TYMV PRO/DUB catalytic dyad remains highly dynamic and its side chains are still often misaligned and their crucial H-bond broken (Fig 2A, bottom). In other cysteine DUBs, that all harbor a third catalytic residue, the catalytic histidine is stabilized thereby. It may still occasionally swing out (*e*.*g*. Fig 8B and S5A Fig, bottom right) but this is associated with an inactive configuration, because the histidine then points away from the other two residues of the triad [46]. Thus the peculiarly mobile catalytic dyad of the TYMV PRO/DUB active site may reflect a need to be further stabilized and/or completed by binding of partners. This too is a well known layer of regulation for cellular DUB activity in several DUB families, as in USP7 for instance, the conformational change of the switching loop being triggered by accessory protein domains [47].

### Two orthogonal strategies for uncoupling TYMV PRO and DUB activities

All DUBs characterized so far recognize the body of Ub through interaction patches distant from their catalytic sites. Mutation of these distant patches has allowed the selective disruption of DUB activity in viral PRO/DUB enzymes from EAV, SARS-CoV and MERS-CoV [22, 43, 44, 48]. Such a strategy also proved successful in the case of TYMV PRO/DUB, as we show here (Table 2 and Fig 4) that mutants of the distant patch centered on I847 are indeed selectively defective in DUB activity.

However, we now demonstrate that uncoupling of PRO from DUB activity can also be achieved by a second, orthogonal strategy, as we show here that the peculiar GPP loop of *Tymoviridae*, which lies right at the entrance to the catalytic cleft, is actually a flap involved in switching from PRO to DUB activity. In turn, mutants interfering with flap closure display phenotypes that are selectively defective in DUB activity. We thus conclude that loop mobility allows alternative recognition of different sequences on the P side, and likely (in)activation of the catalytic site itself. In this scheme, the closed flap conformation is associated with DUB activity, the intermediate conformation with PRO activity, and the open conformation with an inactive PRO/DUB, as borne out by our previous structure of a PRO:PRO complex [21].

The observation that Ile847 patch mutants lack a PRO activity phenotype is somewhat puzzling, as this distant patch is also used by the cleaving PRO to recognize the cleaved PRO [21]. Thus, the Ile847 patch seems less critical for PRO than for DUB activity. This may be linked to the fact that the cleaving PRO also uses two other distant patches (borne by its N-terminal lobe) to recognize its substrate, thus possibly making the Ile847 patch less central to recognition of the PRO substrates. In contrast, recognition of Ub mainly involves a single distant patch recognizing the Ub surface centered on Ile44. While this distant patch differs in different DUBs, all published work reveals that patch disruption impairs DUB activity and could lead to the uncoupling of PRO/DUB dual activities.

### Impairment of the DUB enzymatic activity impacts viral RNA accumulation, whereas the conformation of the GPP flap affects symptoms appearance *in planta*

Although hijacking of the cellular ubiquitin system by viral DUBs is an emerging theme in RNA virus / host interactions, TYMV remains unique to date as an example where the contribution of a viral DUB to the efficiency of viral RNA replication in infected cells has been demonstrated, both in single cells and in whole organisms.

Using two different approaches to assess the contribution of DUB activity to viral replication, i.e. mutating the catalytic cysteine of TYMV PRO/DUB and providing *in trans* cleaved protein products [32], or identifying a set of mutations that uncouple the PRO and DUB activities (this work), we consistently observed that complete or severe loss of DUB activity leads to a ~3- to 4-fold decrease in viral replication efficiency (Fig 6A and 6B), whereas mutants that retain partial DUB activity display intermediate levels of viral RNA accumulation. While traces of intermediate cleavage products were occasionally detected for 98K mutants in the PRO *trans*-cleavage assays (Fig 5), the possible contribution of partially impaired 206K cleavage to the viral replication deficiency was ruled out by the analysis of viral replication proteins produced upon infection (Fig 6C and 6D), as only fully mature proteins were detected under such conditions. Interestingly, a similar effect of DUB activity impairment on viral RNA accumulation was also observed in whole plants (Fig 7B). We thus conclude that TYMV DUB activity plays a critical role in the viral RNA replication process, both in single cells and *in planta*. This finding contrasts with results obtained upon mutation of the OTU DUB domain encoded by the Crimean Congo Hemorrhagic Fever virus (CCHFV), a negative-strand RNA virus from the *Nairovirus* genus [49], as well as mutations dissociating the PRO and DUB activities of the OTU PRO/DUB domain of EAV arterivirus, which revealed equally efficient replication of WT and DUB EAV mutants [22].

This unique feature of TYMV PRO/DUB may be related to the fact that, in contrast to other viral-encoded DUBs described so far, TYMV PRO/DUB does not possess a general deubiquitylating activity, but instead targets specific substrates, including TYMV 66K polymerase [32]. We previously reported that the level of 66K accumulation affects viral RNA replication [28], and that PRO/DUB activity contributed to its degradation rate [32]. It is therefore possible that the defect in TYMV replication upon DUB activity impairment may be linked directly to the decreased stability of the viral polymerase, which would thus become limiting for replication. Alternatively, tuning the ubiquitylation level of the viral polymerase may also contribute to processes other than regulating its stability. In that respect, the (+)RNA tombusviruses were reported to assemble their membrane-bound replication complexes by hijacking ESCRT (endosomal sorting complexes required for transport) complexes [50], a membrane protein-sorting pathway in which reversible ubiquitylation events of cargo proteins are critical (reviewed in [51,52]). Fine tuning of the ubiquitylation level of tombusvirus polymerase appeared important in that process [53]. Whether TYMV DUB activity contributes to the assembly and/or regulation of the membranous spherule-like structures that host the TYMV replication complexes [54] remains to be determined. Finally, it is also conceivable that other, as yet unidentified, substrates of TYMV PRO/DUB may contribute directly or indirectly to the replication deficiency of TYMV DUB mutants.

In addition to the impact of the DUB activity on the extent of viral RNA accumulation, we also observed that mutations affecting the GPP flap had a severe impact on the appearance of symptoms *in planta*. Those ranged from mild chlorosis in the D843A mutant, to local and systemic necrosis in the P866G/P867G mutant and G865A mutants, respectively (Fig 7A). In contrast, the distant Ub-binding patch mutants I847A and I847D, whose GPP flap remains unaffected, displayed chlorotic symptoms similar to a WT infection (Fig 7A).

The symptoms occurring during compatible infections (e.g. chlorotic symptoms) represent the final outcome of complex biochemical and physiological perturbations occurring upon replication and spread of the virus, and result from both the combination of virus demands, cellular stresses and host defense reactions [55]. Despite recent progress in deciphering plant/virus interactions using systems biology approaches [56–59], the molecular mechanisms involved in disease symptoms still remain unclear.

In contrast, much informations have been gained over the years on the mechanisms occurring during incompatible reactions, in which plant immune reactions lead to resistance processes against viral infection. One of the most studied mechanism is that of resistance (*R*) gene-mediated defense responses, also referred to as effector-triggered immunity (ETI) [40, 60], in which a R protein of the plant specifically senses an effector protein encoded by the pathogen. This specific recognition triggers a range of fast and multilayered defense responses, including massive ion fluxes, production of reactive oxygen species, changes in hormonal balance and a massive reprogramming of genome expression [61]. ETI often culminates with the occurrence of a hypersensitive response (HR), a physiological response manifested by local plant cell death, and containment of the pathogen to the initial infection area [39, 40, 60]. Although systemic necrosis is much less characterized than HR, both responses share similar phenotype and molecular features [62], and systemic necrosis is thought to be caused by an incomplete resistance, due to late or weak interaction between the R protein and the elicitor, and not sufficiently strong to restrict virus propagation [63].

In the case of TYMV, the differences in symptoms severity among the various DUB mutants do not correlate with the DUB activity *per se*, but instead seemed to correlate with the conformation adopted by the GPP flap as determined by the molecular dynamics simulations (Fig 2). On one hand, the D843A mutant, whose flap was shown to stabilize in a "closed" conformation more frequently than the WT, led to milder symptoms. On the other hand, the P866G/P867G and G865A mutants, which both appeared unable to properly close their hydrophobic zipper and remained in an "open" or "half-open" position, led to rapid or delayed necrosis, respectively.

Therefore, the appearance of necrotic symptoms upon inoculation of those two mutants may relate to the existence in plant cells of a R protein specifically detecting the "open" conformation of the GPP loop, whose sensing of the P866G/P867G mutant would be strong enough to induce HR and restrict the infection to the viral entry point, whereas part-time or weaker interactions with G865A would induce a delayed systemic necrosis. Transition from HR to systemic necrosis upon point mutation of a viral protein has indeed previously been reported [64]. At present, we also cannot rule out the possibility that other processes than HR-related responses may be involved in the appearance of necrotic lesions, such as the involvement of a general stress response possibly linked to toxicity of the mutant viral proteins, or a premature senescence pathway in response to viral infection. Future experiments will aim at characterizing those processes in more details.

Conversely, because regulators of ETI or HR have been described [65–69], it can also be speculated that the PRO/DUBs bearing a WT GPP flap (i.e. the WT protein, and the I847A and I847D mutants), or a GPP flap with a higher probability to be in a "closed" conformation (i.e. the D843A mutant), may be capable of actively inhibiting cell death/defense responses through specific interaction/sensing of the "closed" flap conformation by some of these regulators. Such a mechanism may as well possibly explain the milder symptoms observed in the D843A mutant.

In that respect, it is interesting to note that many reports support the involvement of DUBs encoded by human- or animal-infecting RNA viruses in counteracting innate immune signaling by various strategies [22, 24, 43, 70–73]. Determining the possible links between TYMV DUB and plant signaling cascades involved in antiviral immunity is an interesting question that the tools developed in the frame of this study will allow us to investigate in the future. Indeed, the TYMV mutants described herein, in which PRO and DUB activities are uncoupled, provide excellent tools for further study of the role of DUB in viral pathogenicity. Searching for additional substrates/partners of TYMV PRO/DUB, as well as system-wide analysis of the transcriptomics or ubiquitylation networks that are linked to viral infection can now be envisaged both at the cellular and whole plant level, which should help clarify the possibly ambivalent role that reversible ubiquitylation events may play during viral life cycles, being either a viral strategy to enhance infectivity, or a host defense reaction, or a combination of both [15]. We expect such studies to provide mechanistic insights into the complexity of virus infection but also to reveal how these mechanisms could be exploited to target pathogenic viruses.

## Materials and methods

### Plasmid constructions

All DNA manipulations were performed using standard techniques [74, 75]. The overall structures of all plasmids were confirmed by restriction analysis, and the sequences of PCR-generated DNA fragments were confirmed by DNA sequencing. The bacterial expression vector encoding the WT PRO/DUB domain of TYMV (residues 728–879 of 206K protein, fused with an N-terminal hexahistidine tag), as well as mutants I847A and I847D, were described previously [21]. Mutants P866G/P867G, G865A, D843A were generated by using a quick Change II site directed Mutagenesis kit (Agilent). Plant expression vectors pΩ-66K, pΩ-98K, pΩ-98K-C783S, pΩ-206K-C783S, and pΩ-(myc)2-Ub were described previously [28, 31, 54, 76]. Plasmids pΩ-98K-P866G/P867G, pΩ-98K-G865A, pΩ-98K-D843A, pΩ-98K-I847A and pΩ-98K-I847D were generated by site-directed mutagenesis of plasmid pΩ-98K, or were obtained from Shanghai ShineGene Molecular Biotech, Inc. (Shanghai, China). *In vitro* infectious transcripts were derived from the full-length TYMV cDNA clone E17 [27], into which the corresponding mutations were introduced by subcloning.

### Protein expression and purification

Recombinant PRO/DUB proteins were produced and purified as described previously [21] with minor modifications. For each construct, an overnight culture of *Escherichia coli* strain Rosetta (DE3) cells (Novagen) was diluted in Luria-Bertani (LB) medium containing 100 μg ml^−1^ ampicillin and 34 μg ml^−1^ chloramphenicol, and grown at 30°C to an optical density at 600 nm (OD^600^) of 0.6. Protein expression was induced by adding isopropyl β-d-thiogalactopyranoside (IPTG) to a final concentration of 0.25 or 0.5 mM, and the cultures were grown at 18°C for 16h. Cells were harvested by centrifugation, washed in PBS buffer and harvested again. The cell pellet was resuspended in lysis buffer (100 mM Tris-HCl pH 7.5, 350 mM NaCl, 25 mM imidazole, 1 mM DTT, 0.5% Triton X-100, 2 mg/ml Lysozyme, and 1 U/ml Benzonase). The mixture was then incubated at 4°C under gentle agitation for 60 min. Lysis was completed by 5 freeze/thaw cycles (70 K/303 K). The mixture was then centrifuged at 35000 × *g* for 30 min or 24000 x *g* for 1h at 4°C to remove insoluble cell debris. The supernatant was purified at 4°C by affinity chromatography using a pre-packed 5 mL His-trap column (GE-Healthcare). The column was first equilibrated with buffer A (100 mM Tris-HCl pH 7.5, 350 mM NaCl, 25 mM imidazole, 1 mM DTT). The supernatant was loaded into the column at a flow rate of 0.5 mL/min. The column was washed with 50 mL of buffer A, followed by 10 mL of washing buffer A1 (100 mM Tris-HCl, pH 6.0, 350 mM NaCl, 25 mM imidazole, 1 mM DTT). The protein was eluted using elution buffer B (100 mM Tris-HCl, pH 7.5, 350 mM NaCl, 500 mM imidazole, 1 mM DTT). Fractions were analyzed on 16.5% Tris-Tricine SDS-PAGE, and those containing PRO/DUB were concentrated using a centrifugal filter device with a 3 kDa cut-off. The concentrated fraction (usually 500 μl) was further purified at 4°C through a high-resolution Superdex S-75 gel filtration column (GE-Healthcare) with Buffer C (10 mM Tris-HCl pH8, 350 mM Ammonium Acetate, 1mM DTT) at a flow rate of 0.25 ml/min. Fractions were analyzed on 16.5% Tris-Tricine SDS-PAGE, and those containing purified PRO/DUB were concentrated. Protein concentration was estimated from the calculated extinction coefficient at 280 nm. WT and mutant PRO/DUB proteins were frozen in liquid nitrogen and stored at –80°C.

### Crystallization and data collection and processing

The detailed protocols for (cross)-seeding-based crystallization of mutants I847A and ΔC5 described elsewhere [38] were used here with some modifications. Briefly, as before, TYMV PRO/DUB ΔC5 and I847A single crystals were obtained by seeding from multiple microcrystals initially obtained for I847A. Microseeding was performed in hanging drops prepared from equal volumes of protein solution in buffer 0.01 M Tris–HCl pH 8, 0.35 M ammonium acetate, 1 mM DTT and reservoir solution. The reservoir solution was composed of 0.1 M trisodium citrate pH 5.6, 0.2 M ammonium acetate, 15%–19%(w/v) PEG 4000, 5%(v/v) MPD. Crystals were transferred for ~5 min in well solution supplemented with 5 mM DTT. Subsequently, the crystals were transferred for ~1 min in well solution supplemented with a mixture of cryoprotectants, as described [77]. For ΔC5, this was cryomix C5 (final concentrations of cryoprotectants 5% diethylene glycol, 10% ethylene glycol, 5% MPD, 5% 1,2-propanediol, 5% glycerol, 5 mM NDSB-201) and for I847A C6 (5% ethylene glycol, 10% MPD, 5% 1,2-propanediol, 5% DMSO, 5% glycerol). Hence, both cryoprotecting solutions contained significant amounts of sulfur-containing compounds (NDSB-201 and DMSO, respectively). Crystals were flash-cooled by plunging into liquid nitrogen. Data were collected on synchrotron SOLEIL beamline Proxima1 and processed with XDS [78].

### Structure determination

The structures were solved by molecular replacement using the PHENIX suite of programs [79]. Rebuilding was performed with Coot [80]. Refinement involved two TLS groups (one for each molecule). Initial noncrystallographic symmetry restraints were removed in later stages of refinement.

### Molecular dynamics simulations and structure visualization

Molecular dynamics simulations of a crystallographic monomer (residues 732–879 of chain A of PDB 4a5u) and of mutants were performed using the AMBER16 program suite [81] with the ff14SB force field. The LEaP program was used for systems' preparation. Hydrogen atoms were added with default parameters, implying that the Nδ1 and Nε2 nitrogens of H869 are protonated and deprotonated, respectively, as desired. Proteins were neutralized with 5 K^+^ cations (4 K^+^ for D843A) and immersed in an explicit TIP3P water box with a solvation shell at least 12 Å-deep. The systems were then minimized and used to initiate molecular dynamics. All simulations were performed in the isothermal isobaric ensemble (*p* = 1 atm, *T* = 300 K), regulated with the Berendsen barostat and thermostat [82], using periodic boundary conditions and Ewald sums for treating long range electrostatic interactions [83]. The hydrogen atoms were constrained to the equilibrium bond length using the SHAKE algorithm [84]. A 2-fs time step for the integration of Newton’s equations was used. The nonbonded cutoff radius of 10 Å was used. All simulations were run with the SANDER module of the AMBER package.

All simulation trajectories and crystal structures were visualized and structural figures were made with PyMOL [85]. PyMOL was also used to introduce mutations as needed for simulations prior to system preparation.

### Deubiquitylation assay *in vitro*

Prior to deubiquitylation assay, purified proteins were dialyzed overnight at 4°C in buffer 50 mM HEPES-KOH pH 8.0, 150 mM KCl, 1 mM DTT, 10% glycerol, adjusted to a concentration of 100 μM and kept at –80°C until use. The fluorogenic substrate Ub-AMC (Boston Biochem) dissolved in DMSO was diluted in assay buffer (50 mM HEPES-KOH pH 7.8, 10 mM KCl, 0.5 mM EDTA, 5 mM DTT, 0.5% Nonidet-P40). DUB activity was assessed at room temperature in a Hitachi F2000 spectrofluorometer in assay buffer with a final concentration of DMSO adjusted to 2% to match the DMSO concentration in the highest Ub-AMC concentration assays. Reactions were initiated by the addition of enzyme to the cuvette and the rate of substrate hydrolysis was determined by monitoring AMC-released fluorescence at 440 nm (excitation at 380 nm) for 10 min. Enzyme concentrations for each mutant were 125 nM for WT PRO, 500 nM for P866G/P867G, G865A, D843A and I847A, and 1 μM for I847D, respectively. In order to determine the apparent *k*_cat_ / *K*_m_ (*K*_app_), the substrate concentration was kept at a concentration below 0.5 μM, where the initial velocity is linear with substrate concentration, and *K*_app_ values were then determined according to the equation V / [E] = *K*_app_ / [S] as described previously [32]. Depending on the batch of Ub-AMC, the WT enzyme displayed high variability, with *K*_app_ varying between 1593 ± 82 M^–1^s^–1^ and 3855 ± 170 M^–1^s^–1^. Hence, the activity of the WT protein was measured as a reference for each independent experiment, and *K*_app_ of each of the mutant proteins was normalized to that of the WT protein measured simultaneously. All experiments were performed at least in duplicate, and data were expressed as the means and standard deviations of these independent experiments.

### Preparation and transfection of Arabidopsis protoplasts

Protoplasts of *Arabidopsis thaliana* were prepared and transfected with 4 to 15 µg of plasmids or *in vitro* transcripts as described previously [28], with minor modifications [86]. Where applicable, samples were supplemented with the control vector pΩ-REL encoding Renilla luciferase [28] to keep the total amount of nucleic acids transfected constant. Capped *in vitro* transcripts were generated from linearized DNA templates as described previously [27]. Each viral mutant was transfected in Arabidopsis protoplasts between 7 and 12 times in at least two independent experiments using various batches of *in vitro* transcripts.

### Inoculation of Arabidopsis plants

*Arabidopsis thaliana* plants (ecotype Col-0) were grown at 19–21°C under a 16 h light/8 h dark photoperiod in a growth chamber, and plants were inoculated ~ 5–6 weeks after seed germination, before the occurrence of floral transition. The inoculum consisted of capped *in vitro* transcripts (4.5 μg per plant) diluted in 45 μl of water. Plants were mechanically inoculated by rubbing the upper surface of three rosette leaves using celite as an abrasive as previously described [87, 88]. After inoculation, the plants were allowed to grow in the same conditions and were monitored regularly for symptom development. Photographs of the inoculated leaves were taken at 14 days post-inoculation (dpi), and those of systemic leaves (i.e. leaves distant from the inoculated leaves) were taken at 17 dpi. Plants were harvested at 17 dpi, frozen in liquid nitrogen and stored at –80°C. Each viral mutant was inoculated to a total of 26 to 32 plants, in at least four independent experiments using various batches of *in vitro* transcripts.

### Antibodies, immunoprecipitation and immunoblotting experiments

Total protein extraction from protoplasts, immunoprecipitation (IP), SDS-PAGE, immunoblotting and detection of viral proteins were performed as described [31, 54, 77, 89] using either NBT/BCIP or CDP-Star as a substrate. Polyclonal antisera raised against the TYMV 66K protein, the PRR domain of 98K protein (hereafter anti-98K antiserum) and the TYMV capsid were described previously [54, 89], and were used at dilutions of 1/2,000, 1/8,000, and 1/50,000, respectively.

Detection of 66K-Ub conjugates following 66K IP was performed as described [28, 32]. Immunoblotting with anti-*myc* was performed to detect *myc*-tagged ubiquitylated proteins using CDP-Star as a substrate (Roche) followed by anti-66K to verify levels of 66K. Anti-*myc* monoclonal antibody (9E10) (Roche) was used at 1/3,000 dilution.

### RNA isolation and cDNA synthesis

Transfected protoplasts were collected 48h post-transfection by centrifugation at 80 x *g*, immediately frozen in liquid nitrogen and stored at –80°C. Plant samples were collected at 17 dpi, immediately frozen in liquid nitrogen and stored at –80°C. Frozen tissue were ground to a fine powder using a Retsch Mixer Mill MM400 and ~ 60 mg of material was used for RNA extraction. Total RNA extraction was performed as previously described [90] using Trireagent (MRC), and the concentration and purity of the RNA samples was determined using a Nanodrop spectrophotometer. Following quantification, all RNA samples were normalized to 200 ng/μl, and 500 ng of total RNAs were then treated with DNase I (ThermoFisher) and reverse-transcribed using random hexamer primers (5 μM) and Revertaid Premium Reverse Transcriptase or Maxima H-minus Reverse Transcriptase (ThermoFisher) in the presence of Ribolock RNase inhibitor (ThermoFisher) according to the manufacturer's instructions. cDNA synthesis was confirmed by PCR amplification of EFIα cDNA using gene-specific primers (Table 3), and verification by agarose gel electrophoresis for the presence of a product of appropriate size.

**Table 3:** Primers used for RTqPCR experiments.

primer name	target gene	nucleotide sequence	Tm (°C)	amplicon size (nt)	PCR efficiency in protoplast RNA samples	PCR efficiency in plant RNA samples
TYMV 150–30 TYMV 150–31	TYMV genomic RNA	CAACTGGTCTTATACCCACTTCCG CGGAATGGACCATGGGTAGG	65.2 62.5	191 nt	94.1%	100.7%
EF1 F2 EF1 R1	*EF1α (*At5g60390)	TGGTAACGGTTACGCCCCA ACCAGCGTCACCATTCTTCAAA	59.5 60.3	142 nt	94.5%	97.8%
PDF2 F5 PDF5 R5	*PDF2 (*At1g13320)	AGTGAGAACAATGACGATGACG GCATACTCAACCCCTCCTACATAC	60.3 65.2	92 nt	91.6%	not done
18S F 18S R	*18S* rRNA	GGGAAACTTACCAGGTCCAGACATAG CCACGTAGCTAGTTAGCAGGCTGAG	67.9 69.1	159 nt	not done	102.9%

### qPCR amplification and quantification of viral RNA accumulation

qPCR reactions were performed in 384-well plates with a LightCycler 480 Real-Time PCR system (Roche). Reactions were in 10-μl volumes containing 1 ng of cDNA in the case of protoplast RNA samples, or 0.1 ng of cDNA in the case of plant RNA samples, 500 nM of each primer and HOT FIREPol EvaGreen qPCR Mix Plus (Solis BioDyne, Estonia). An epMotion automate (Eppendorf) was used to dispense reagents, and each qPCR reaction was performed in three replicates. Cycling parameters consisted of a preincubation step at 95°C for 15 min, and 40 amplification cycles of denaturation at 95°C for 15 s, primer annealing at 60°C for 15 s, and elongation at 72°C for 30 s. Fluorescence values were acquired at the end of the elongation phase. Amplification was followed by a single melting-curve analysis cycle between 60°C and 95°C with five fluorescence acquisitions per degree.

Primers for qPCR amplification of the TYMV genome and reference genes were designed using the software Primer 3 Plus. The efficiency of amplification for each primer pair was determined using serial dilutions of cDNA templates prepared from infected protoplasts or infected plants, respectively, and linear regression from standard curves generated via LightCycler 480 analysis (Table 3). Relative quantities of cDNAs were calculated and normalized as described [91, 92]. According to previous validation analyses [93], the Arabidopsis reference genes chosen to normalize data from protoplast samples were *EF1α (*At5g60390) and *PDF2 (*At1g13320). In the case of plant samples, the abundance of viral RNAs precluded the use of *PDF2*, and the reference genes chosen to normalize data were *EF1α* and *18S* rRNA as previously described [94]. Replicate samples with a quantification cycle value (Cq) differing from the mean Cq value by more than 2 units were considered as outliers and were excluded from the calculation. Data relative to mutant transcripts were then expressed as a percentage of the mean value of the data obtained with WT transcripts that were transfected or inoculated simultaneously and analyzed by qPCR in the same run (S1 Dataset).

## Supporting information

S1 Fig Schematic representation of the TYMV 206K polyprotein. Domains indicative of methyltransferase (MT), proteinase/deubiquitinase (PRO/DUB), helicase (HEL) and polymerase (POL) activities are indicated. 206K protein is processed proteolytically at peptide bonds 879–880 (PRO↓HEL) and 1259–1260 (HEL↓POL) indicated by dashed lines, to release mature viral proteins of 98K, 42K and 66K [31].
(TIF)

S2 Fig Temperature factors along the TYMV PRO/DUB sequence according to experiment and simulations. (A) Temperature factors of the 'A' and 'B' molecules in the ΔC5 crystal. The 'B' molecule (green curve) is less well ordered than the 'A' molecule (blue curve), except around residue L820 and at loop 864-TGPPS-868. (B) RMS fluctuations of the backbone during 25-ns molecular dynamics simulations for wild-type, full-length PRO/DUB and for mutants interfering with mobility of loop 864-TGPPS-868. The wild type backbone dynamics match the temperature factors of the ΔC5 'A' molecule well, while the mutants display higher mobility of the loop.
(TIF)

S3 Fig Final electron density maps 2mFo-DFc (blue, 1 sigma contour) and mFo-DFc (green, 5 sigma contour) of the active sites of molecules 'A' and 'B'.
(TIF)

S4 Fig Overlay of the asymmetric units of crystals of ΔC5 and I847A. Color code as in Fig 2. No fitting was performed.
(TIF)

S5 Fig Layouts of the two sides of the active site clefts in the coronavirus USP-like PRO/DUBs in the same representation as for the OTU DUBs of Fig 8. (A) The SARS CoV PRO/DUB in free (PDB 2FE8) and diubiquitin-bound (PDB 5E6J) forms. Note the large size and displacement of the glycine-hinged loop bearing C271 between the two forms. (B) The MERS CoV PRO/DUB in free (PDB 4REZ) and ubiquitin-bound (PDB 4RF0) forms. Top left panel: Here the glycine-hinged loop is disordered in the free form. The asterisks denote G1752 and G1758 on either side of the disordered loop.
(TIF)

S6 Fig Layouts of the two sides of the active site cleft in the USP7 cellular DUB in a similar representation as for the OTU DUBs of Fig 8. The switching loop is located by residue Q293 and labeled. In the bottom panels, the distances between the sulfur of C223 and the Nδ1 of H464 are indicated as in Fig 1B. Left, free USP7 (PDB 1NB8). Right, ubiquitin-bound USP7 (PDB 1NBF).
(TIF)

S1 Dataset RT-qPCR raw data, quantification and normalization, and relative RNA accumulation of mutant transcripts in Arabidopsis protoplasts and Arabidopsis plants. (XLSX)

S2 Dataset Original dataset for Fig 4. (TIF)

S3 Dataset Original dataset for Fig 5. (TIF)

S4 Dataset Original dataset for Fig 6. (TIF)
