# Supplementary figures and images for: A mobile loop near the active site acts as a switch between the dual activities of a viral protease/deubiquitinase
Source: PLoS Pathog. 2017 Nov 8;13(11):e1006714. doi: 10.1371/journal.ppat.1006714 (PMC5695851; doi:10.1371/journal.ppat.1006714)

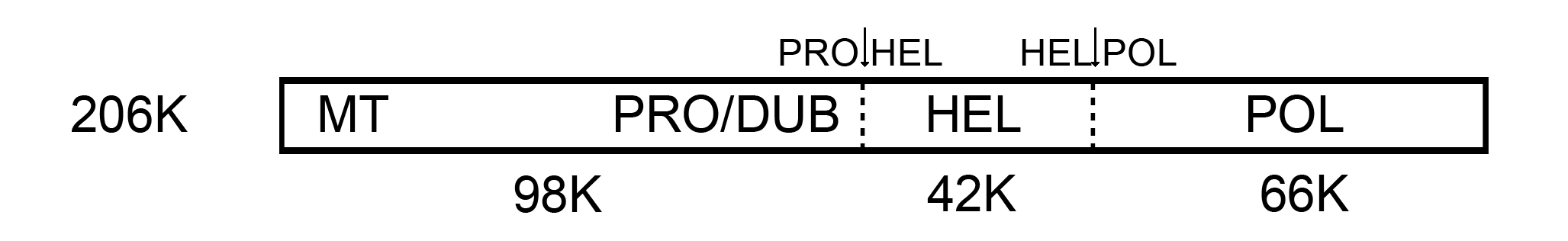

Supplement: S1 Fig — Domains indicative of methyltransferase (MT), proteinase/deubiquitinase (PRO/DUB), helicase (HEL) and polymerase (POL) activities are indicated. 206K protein is processed proteolytically at peptide bonds 879–880 (PRO↓HEL) and 1259–1260 (HEL↓POL) indicated by dashed lines, to release mature viral proteins of 98K, 42K and 66K [31]. (TIF) [file ppat.1006714.s001.tif]

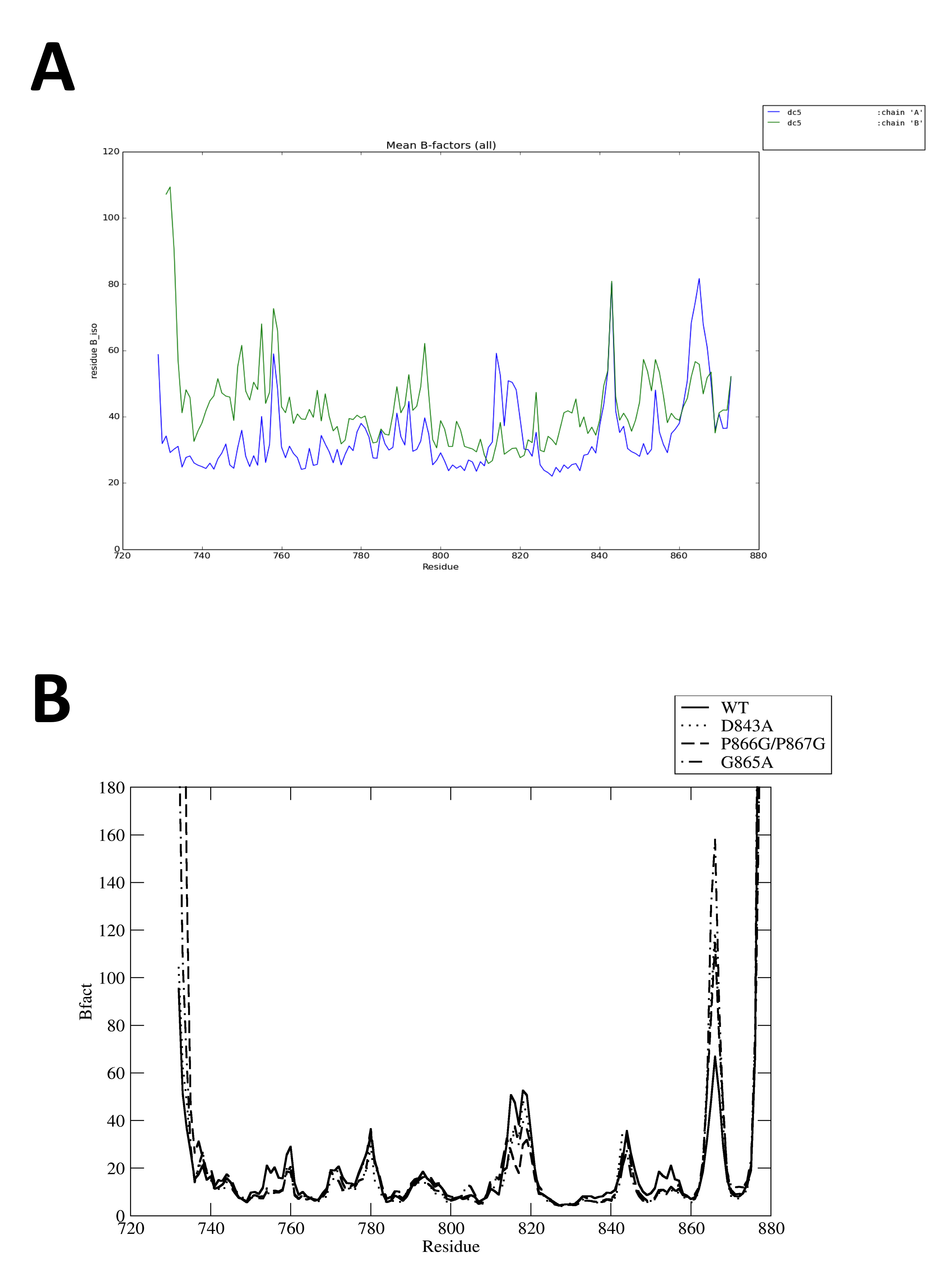

Supplement: S2 Fig — (A) Temperature factors of the 'A' and 'B' molecules in the ΔC5 crystal. The 'B' molecule (green curve) is less well ordered than the 'A' molecule (blue curve), except around residue L820 and at loop 864-TGPPS-868. (B) RMS fluctuations of the backbone during 25-ns molecular dynamics simulations for wild-type, full-length PRO/DUB and for mutants interfering with mobility of loop 864-TGPPS-868. The wild type backbone dynamics match the temperature factors of the ΔC5 'A' molecule well, while the mutants display higher mobility of the loop. (TIF) [file ppat.1006714.s002.tif]

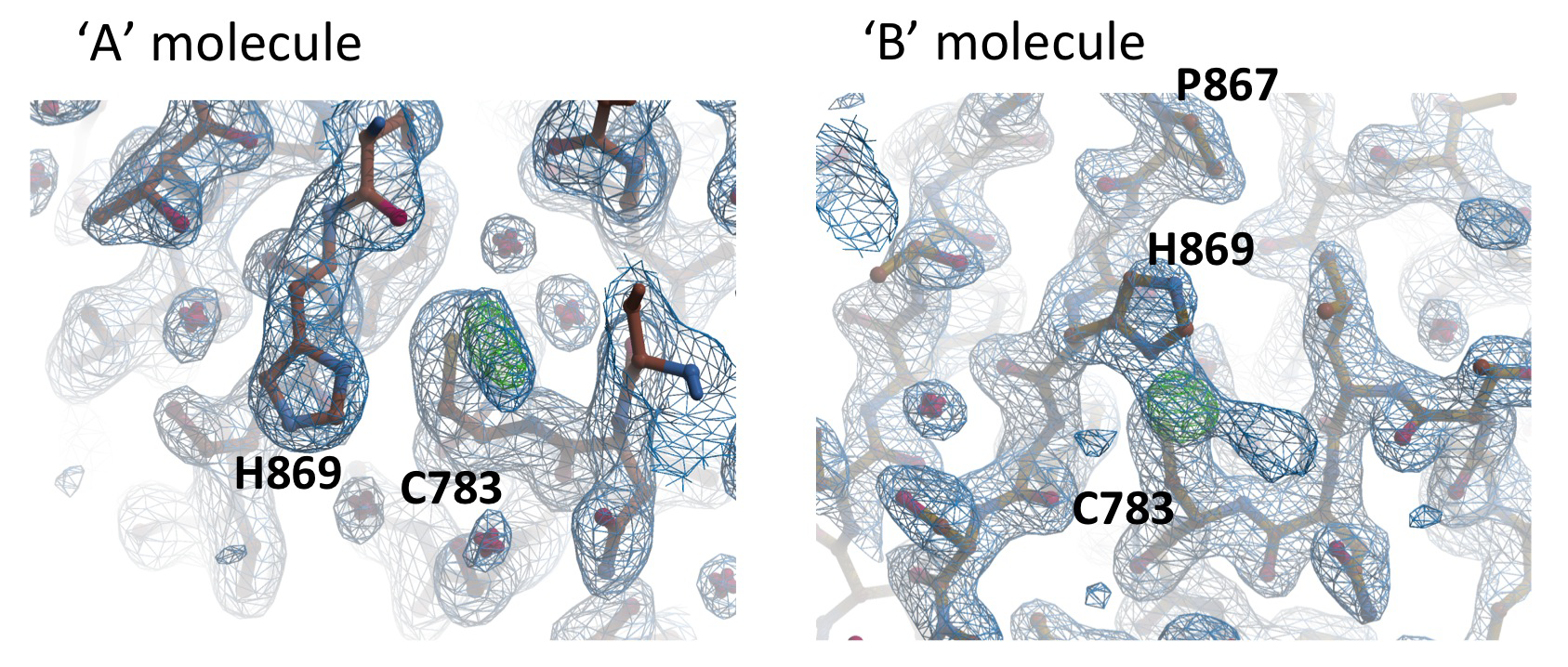

Supplement: S3 Fig — Final electron density maps 2mFo-DFc (blue, 1 sigma contour) and mFo-DFc (green, 5 sigma contour) of the active sites of molecules 'A' and 'B'. (TIF) [file ppat.1006714.s003.tif]

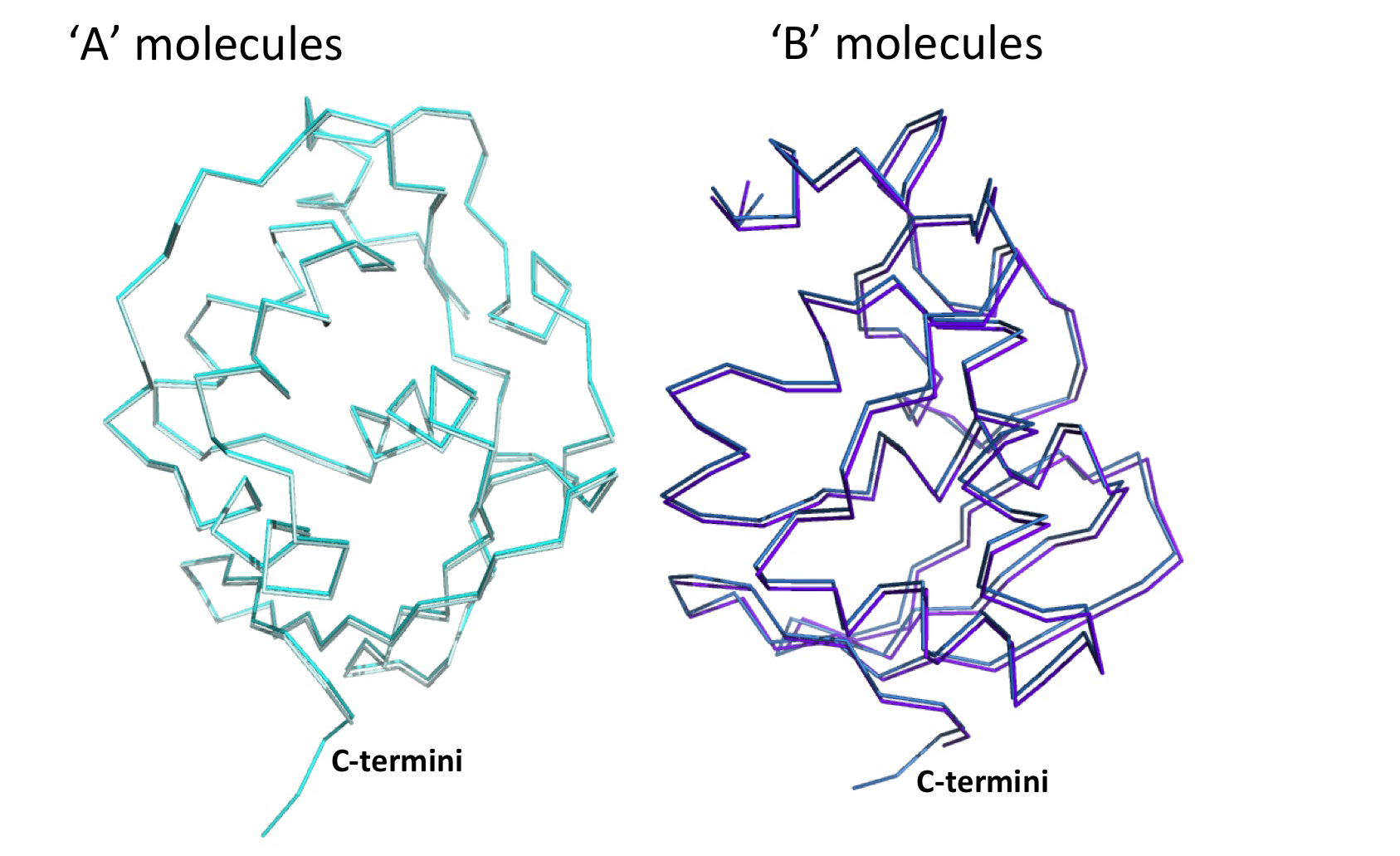

Supplement: S4 Fig — Color code as in Fig 2. No fitting was performed. (TIF) [file ppat.1006714.s004.tif]

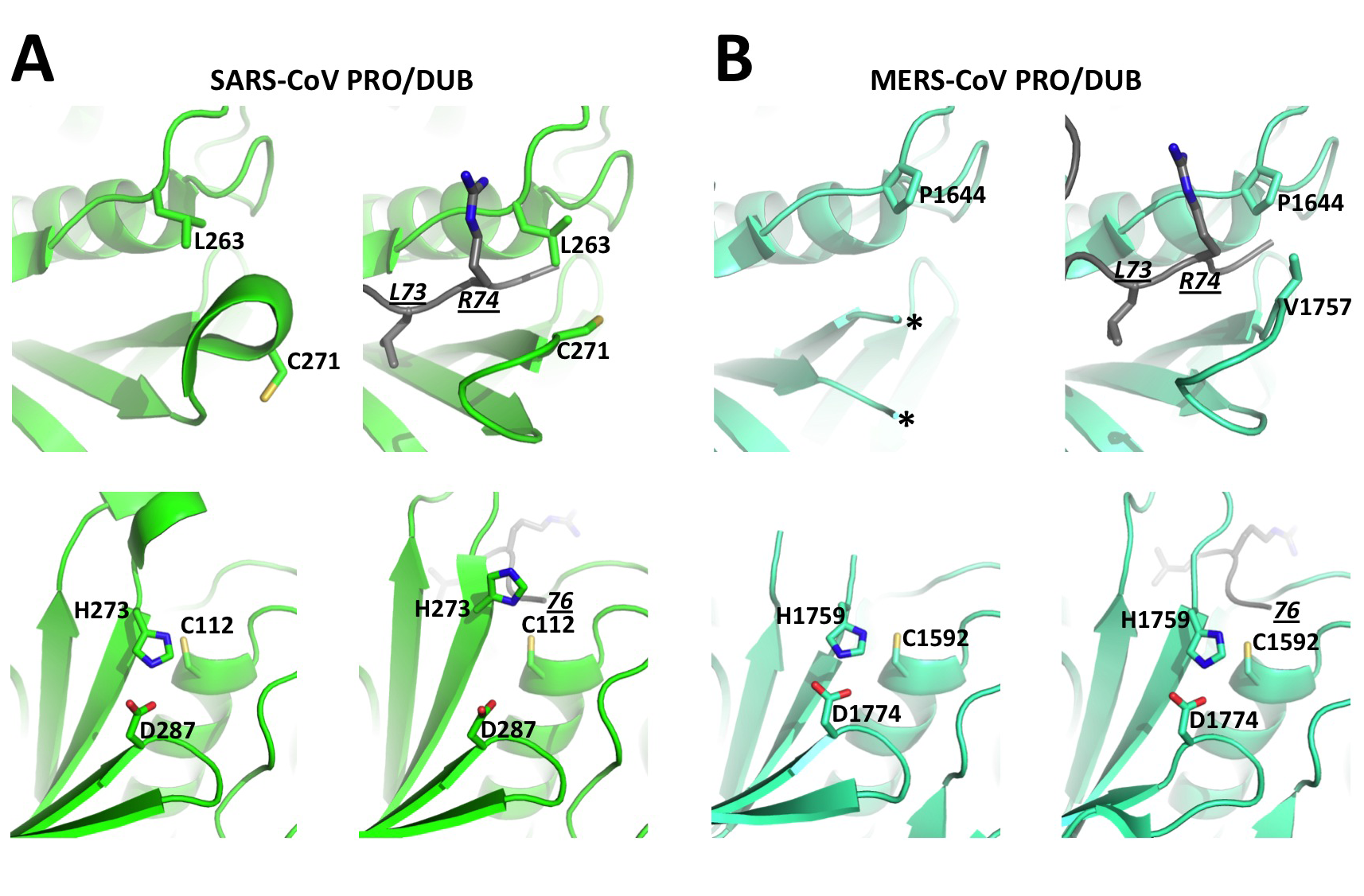

Supplement: S5 Fig — (A) The SARS CoV PRO/DUB in free (PDB 2FE8) and diubiquitin-bound (PDB 5E6J) forms. Note the large size and displacement of the glycine-hinged loop bearing C271 between the two forms. (B) The MERS CoV PRO/DUB in free (PDB 4REZ) and ubiquitin-bound (PDB 4RF0) forms. Top left panel: Here the glycine-hinged loop is disordered in the free form. The asterisks denote G1752 and G1758 on either side of the disordered loop. (TIF) [file ppat.1006714.s005.tif]

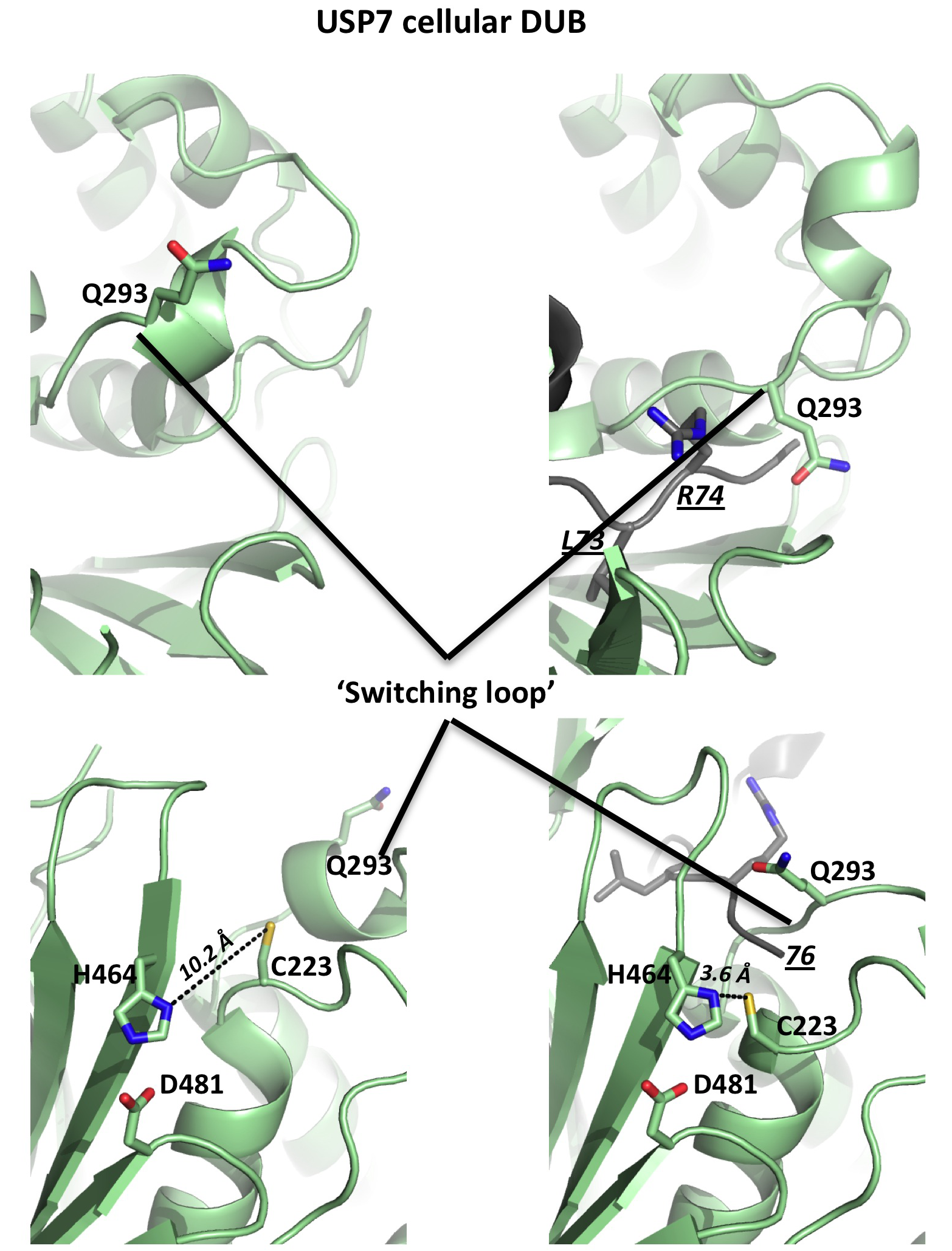

Supplement: S6 Fig — The switching loop is located by residue Q293 and labeled. In the bottom panels, the distances between the sulfur of C223 and the Nδ1 of H464 are indicated as in Fig 1B. Left, free USP7 (PDB 1NB8). Right, ubiquitin-bound USP7 (PDB 1NBF). (TIF) [file ppat.1006714.s006.tif]

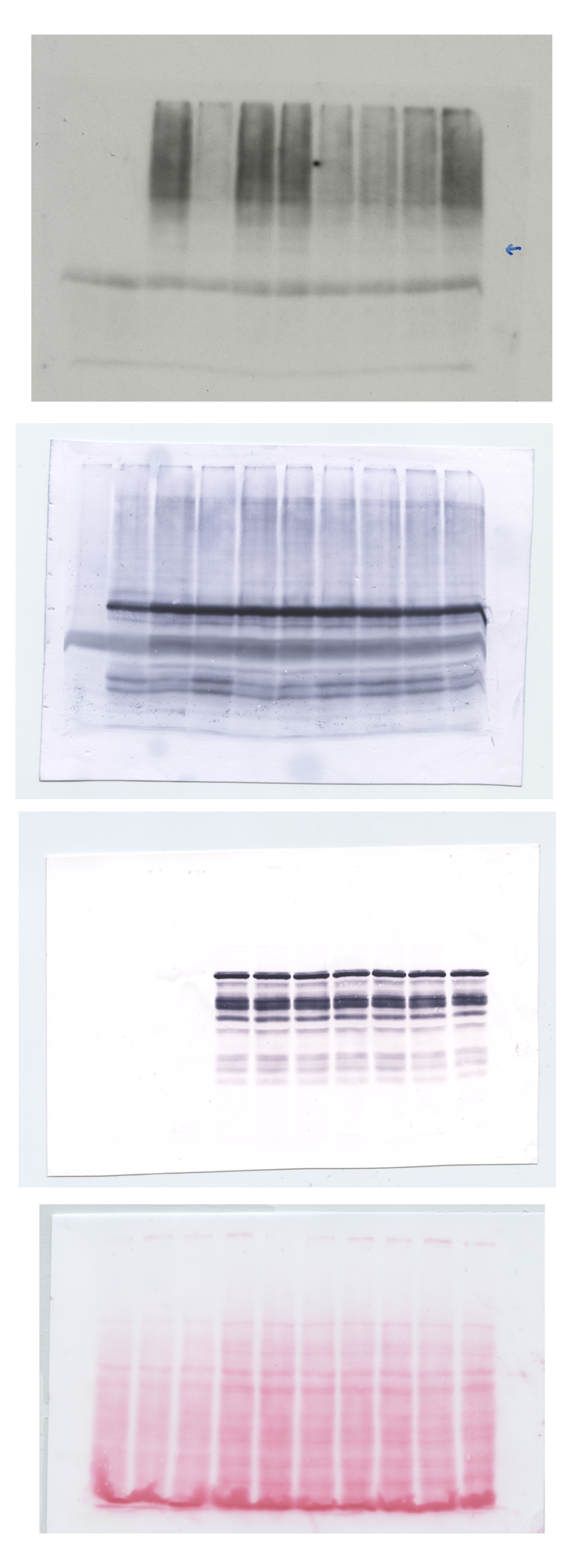

Supplement: S2 Dataset — (TIF) [file ppat.1006714.s008.tif]

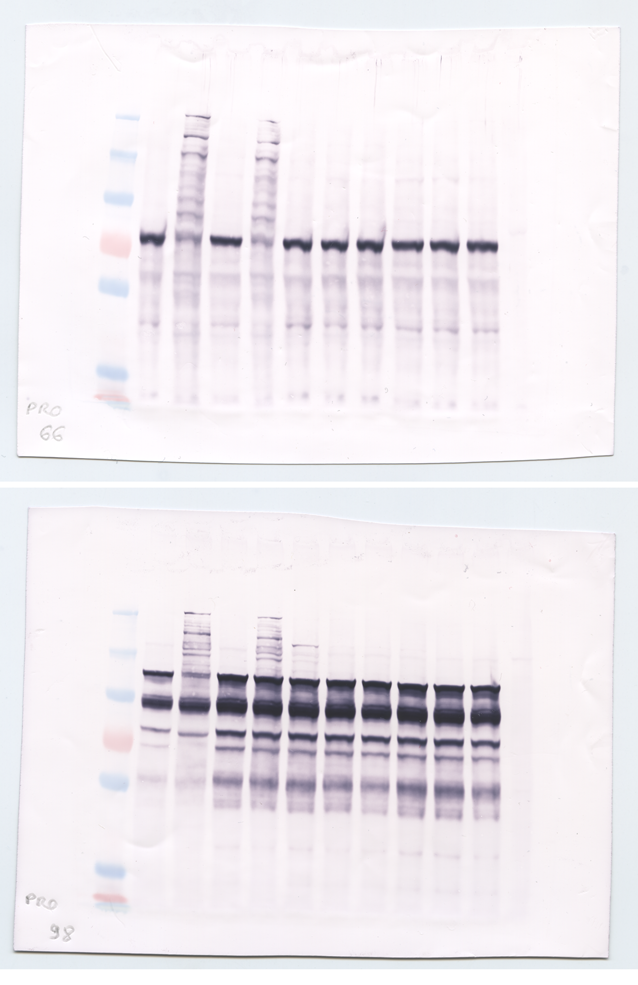

Supplement: S3 Dataset — (TIF) [file ppat.1006714.s009.tif]

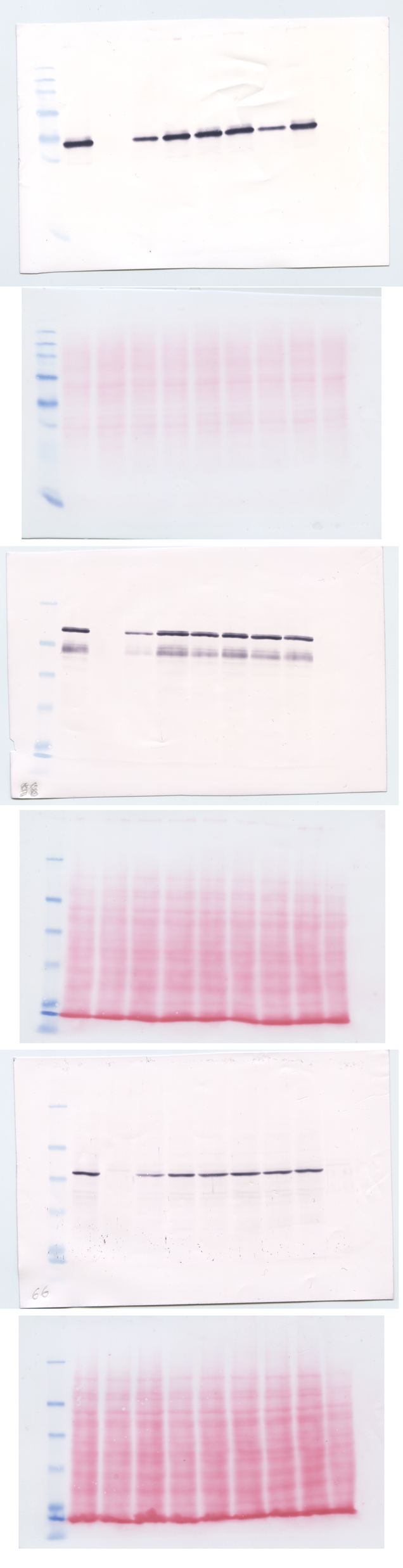

Supplement: S4 Dataset — (TIF) [file ppat.1006714.s010.tif]
